# The association between pressure injury microbiome and wound healing: a systematic review

**DOI:** 10.3389/fcimb.2025.1703418

**Published:** 2026-01-08

**Authors:** Cyra Schmandt, Enkeleda Llukovi, Simona Capossela, Reto Wettstein, Ezra Valido, Magda Gamba, Claudio Perret, Jivko Stoyanov, Alessandro Bertolo

**Affiliations:** 1 SCI Population Biobanking & Translational Research Group, Swiss Paraplegic Research, Nottwil, Switzerland; 2 Graduate School for Cellular and Biomedical Sciences, University of Bern, Bern, Switzerland; 3 Department of Plastic, Reconstructive, Aesthetic and Hand Surgery, University Hospital of Basel, Basel, Switzerland; 4 Central and University Library of Lucerne, Lucerne, Switzerland; 5 Faculty of Health Sciences and Medicine, University of Lucerne, Lucerne, Switzerland; 6 Neuro-Musculoskeletal Functioning and Mobility, Swiss Paraplegic Research, Nottwil, Switzerland; 7 Institute of Social and Preventive Medicine, University of Bern, Bern, Switzerland

**Keywords:** chronic wounds, microbiome, pressure injuries, wound healing, wound management

## Abstract

**Introduction:**

Pressure injuries (PIs) are a significant clinical problem, particularly in elderly, bedridden, and spinal cord injury patients. Bacterial infections are a primary complication that often delays or prevents wound healing. This systematic review analysed the current evidence on the role of the PI microbiome in wound healing outcomes.

**Methods:**

A systematic search was conducted in three online databases, namely Embase, Medline, and Web of Science (latest search October 2024). In total, 20 studies met the inclusion criteria, of which three were interventional (randomised controlled trials (RCTs), n=2; pre-post study, n=1), and 17 were observational study designs (retrospective, n=6; prospective, n=8; and case-control, n=3) comprising 1'015 study participants (with 1'034 PIs). These studies examined the PI microbiome, mostly at PI grades III and IV, using culture-based and next-generation sequencing (NGS) techniques. Data extraction focused on microbial diversity, predominant species, and their association with wound healing. The risk of bias was categorised as moderate, mostly due to the absence of sample size justification, as assessed by the NHLBI tool.

**Results:**

The findings confirmed that Staphylococcus aureus, Pseudomonas aeruginosa, Proteus mirabilis, Klebsiella pneumoniae, Enterococcus spp., and Escherichia coli dominated the PI microbiomes. Microbiome composition varied according to PI severity and anatomical location. Molecular techniques have identified a more diverse microbiome than culture-based methods. Although no specific bacterial taxa have been found to be favourable for wound healing, many taxa were found to be detrimental to PI development, including Anaerococcus, Finegoldia, and Acinetobacter. Antibiotic resistance was common, particularly in S. aureus. Interventions targeting the microbiome, such as debridement and platelet-rich plasma therapy, have been shown to improve healing rates.

**Discussion:**

In conclusion, evidence showed that the composition of the PI microbiome might negatively associate to wound healing, with the dominance of anaerobes associated with delayed healing. Therefore, future PI treatments should prioritise patient-centred approaches that integrate advanced microbial profiling with rigorous clinical evaluation to optimise chronic wound management.

**Systematic Review Registration:**

https://www.crd.york.ac.uk/prospero/, identifier CRD42024575143.

## 1 Background

**Figure 1:**
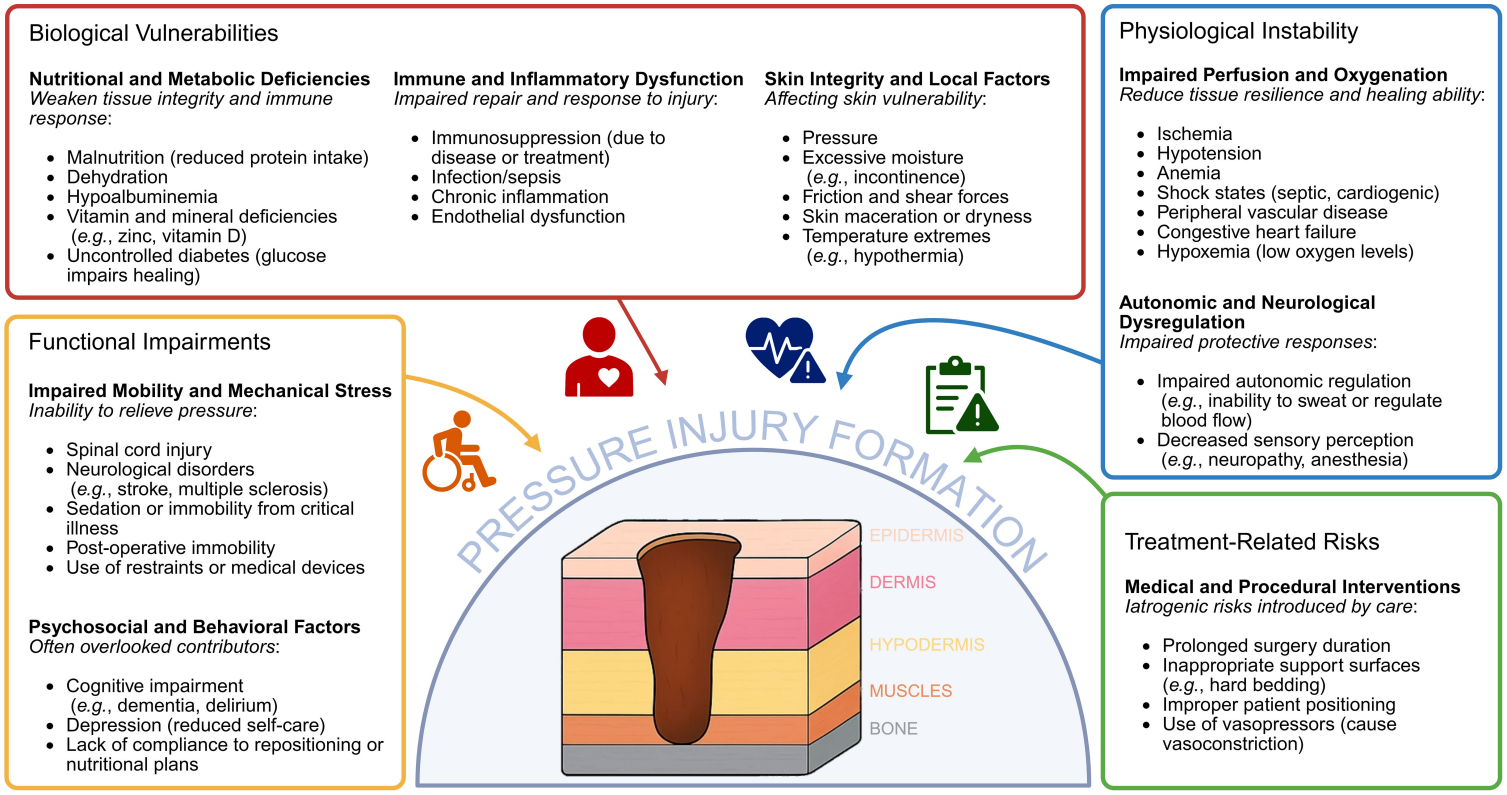
Overview of key risk factors contributing to PI formation and how they interact with host factors relevant to the skin microbiome. The diagram illustrates five major categories of vulnerability: Biological vulnerabilities (nutritional and metabolic deficiencies, immune and inflammatory dysfunction, and local skin integrity factors); Functional impairments (immobility, mechanical stress, and psychosocial/behavioural contributors); Physiological instability (impaired perfusion and oxygenation, autonomic dysregulation, and reduced sensory responses); and Treatment-related risks (medical and procedural factors that increase iatrogenic injury risk). These interconnected factors influence tissue resilience, immune function, and the local microenvironment, collectively shaping susceptibility to PI and modulating host–microbiome interactions at the skin surface. Created in BioRender. Bertolo, A. (2025), https://BioRender.com/g2d7kdj.

Pressure injuries (PIs), also commonly referred to as pressure ulcers, decubitus ulcers, or bedsores, result from prolonged, unrelieved pressure over bony prominences. They can range from intact but reddened skin in grade I PIs, to shallow open sores in grade II, deeper tissue damage reaching the fat layer in grade III, and in the most severe cases (grade IV), open wounds with exposed bone (Mendes et al., 2019). Ischaemia induced by sustained vascular deformation from pressure compromises tissue viability by reducing the delivery of oxygen and nutrients, and removal of potentially toxic metabolites. Mechanical forces, such as compression, tension, and shear, further exacerbate tissue damage. Tissue susceptibility to PI varies depending on the tissue type and is influenced by several factors, including microclimate, perfusion, and existing tissue pathologies which interact in a complex cascade, amplifying tissue damage over time (Bluestein and Javaheri, 2008; Flemming et al., 2016; Huang et al., 2020). Beyond mechanical forces, internal factors such as malnutrition, anaemia, endothelial dysfunction, advanced age, diabetes mellitus, urinary or bowel incontinence, loss of sensibility, and disturbance of autonomic regulation can further increase the risk of PI development (**Figure 1**).

PIs are an important clinical issue and are particularly prevalent among elderly individuals, stroke survivors, patients with diabetes or dementia, and those who are bedridden, wheelchair-bound, or have impaired mobility or pressure sensation (Rappl, 2008). For example, individuals with spinal cord injury (SCI) have an increased risk of developing PIs, and the presence of a PI can escalate healthcare costs four-fold and extend hospitalisation durations six-fold (Stroupe et al., 2011). The clinical and economic burden of PIs is significant; for example, in the United States alone, over 2.5 million hospitalised patients are treated for PIs each year, with annual healthcare costs estimated at $11 billion (Sen et al., 2009). The 2019 Global Burden of Disease study reported approximately 3.17 million cases of PIs and nearly 25,000 related deaths (Ding et al., 2024). Conventional management strategies, including pressure unloading, wound care, negative pressure therapy, debridement, and nutritional optimisation, are generally effective for superficial PIs (grades I and II) (Kandi et al., 2022). However, deeper injuries (grades III and IV) often require surgical debridement and interventions, mainly flap reconstruction or skin grafting for defect closure (Guo and Dipietro, 2010). Despite these efforts, complications such as delayed wound healing, wound dehiscence, haematoma and seroma formation, and infections remain common, prolonging patient recovery and substantially increasing healthcare costs (DeVivo and Farris, 2011).

A primary and debilitating complication of PIs is bacterial infection, ranging from localised wound infection to deep tissue involvement, osteomyelitis, septic arthritis, and bacteraemia (Dana and Bauman, 2015). Osteomyelitis is an infection of the bone tissue that may develop without systemic symptoms and is reported in 17% to 32% of patients affected by grade IV PI (Andrianasolo et al., 2018). Osteomyelitis is often associated with delayed wound healing and dehiscence, whereas bacteraemia typically triggers systemic inflammatory responses. Bacterial infections of PIs are characteristically polymicrobial, harbouring multiple bacterial pathogens often organised within a biofilm phenotype. Biofilms serve as a notable barrier to healing and exhibit intrinsic resistance to antibiotics and host immune responses. The bacterial load is a critical determinant: generally, a density exceeding 10^5^ colony-forming units (CFU) per gram of tissue is considered to inhibit normal wound healing, although some evidence suggests that even lower bacterial counts may retard the process (Sato et al., 2018). Common bacterial culprits identified in PI infections include *Staphylococcus aureus* - often the most prevalent, including methicillin-resistant *S. aureus* (MRSA) - *Pseudomonas aeruginosa*, and members of the Enterobacteriaceae family like *Escherichia coli* and *Proteus mirabilis* (Dana and Bauman, 2015; Gomes et al., 2022). Anaerobic bacteria, such as *Anaerococcus* and *Finegoldia*, are also frequently found, especially in deep infections (Dunyach-Remy et al., 2021). Wound contamination from external sources, such as faeces and urine, which is common in bedridden patients, further exacerbates colonisation by gut- and urine-related bacteria, contributing to infection and delayed healing (Singh et al., 2015). Our previous research found that bacterial clusters containing *Staphylococcus*, *Streptococcus*, and *Proteus* were associated with a higher rate of complications, suggesting that the presence of these bacteria may predict impaired healing (Wettstein et al., 2023). If left untreated, such infections can lead to severe local and systemic complications (Dana and Bauman, 2015). The rise of multidrug-resistant (MDR) organisms, including MRSA and extended-spectrum beta-lactamase (ESBL)-producing Enterobacteriaceae, presents an emerging and complex problem that complicates clinical outcomes and treatment options (Binsuwaidan et al., 2023). The diagnosis of infections and antibiotic resistance relies on clinical examination, microbiological and histopathological assessments, and imaging.

Although widely used, traditional culture-based techniques have limitations in detecting the full spectrum of wound microbiota. Over the past two decades, molecular diagnostic methods, including bacterial tag-encoded FLX amplicon pyrosequencing and 16S rRNA sequencing, have revealed that many predominant organisms in chronic wounds are not detected by standard cultures (Dana and Bauman, 2015; Wettstein et al., 2023). For example, pre-molecular era data suggested that fungi and yeasts were detected in less than 2% of chronic wounds, while molecular analyses have reported fungal presence in 23% of wound samples, illustrating the shortcomings of culture-based methods (Dowd et al., 2011). Advances in deep sequencing techniques have provided new insights into the composition of the wound microbiome and its impact on infection and healing (Kalan et al., 2019). This is particularly important because effective infection control is essential to prevent pathogen dissemination and limit antimicrobial resistance (Livesley and Chow, 2002).

The microbiome composition of PIs typically originates from the surrounding skin tissues (Konya et al., 2005; Nagase et al., 2020; Wettstein et al., 2023), often comprising a mixture of commensal and pathogenic species (Ramsey et al., 2016). However, the contributions of individual microbial species and the overall microbial community to wound healing outcomes remain insufficiently characterised. Despite the growing recognition of the importance of the microbiome, no consensus exists regarding the specific microorganisms or microbial profiles that predict favourable or unfavourable healing trajectories in PIs. Interestingly, not all bacteria hinder the wound healing process. For instance, *Alcaligenes* has been shown to promote re-epithelialisation in diabetic ulcers by downregulating tissue-destructive enzymes (White et al., 2024). This finding highlights the dual role of the microbiome in tissue regeneration and identifies potential therapeutic targets.

Therefore, this systematic review aims to synthesise and critically analyse the existing literature on the role of the PI microbiome in wound healing outcomes. Specifically, this review addresses the following research questions: (i) what is the association between microbial colonisation and the healing trajectory of PIs? (ii) what factors influence this association, and how does the PI microbiome vary depending on infection characteristics? and (iii) how do the anatomical location and severity of PIs affect the wound microbiome?.

## 2 Methods

### 2.1 Data sources and search strategy

The systematic review was conducted following the recommendations of the Collaboration and reported according to the Preferred Reporting Items for Systematic Review and Meta-Analyses (PRISMA 2020) guidelines (**Supplementary Material File 1**) (Muka et al., 2020). The detailed study protocol is available in the PROSPERO database (registration number: CRD42024575143). In brief, the search strategy was elaborated and conducted with the assistance of a specialist librarian guided by a Population Exposure Outcome framework approach which incorporated both subject headings and free-text terms extracted from the titles and abstracts of relevant articles, focusing on the microbiome in PIs (exposure) and ulcer healing (outcome). We systematically searched three databases, namely Embase.com, Medline (via Ovid), and Web of Science (latest search October 2024). We used search terms relevant to the research question, such as “bacteria”, “microbiota”, and “pressure injuries” (complete search strategy in **Supplementary Material File 2**). To identify additional relevant articles, the references of all included studies were hand searched, as well as studies citing the included articles. Search results from all databases were imported into a single EndNote library (EndNote, Clarivate, v. 20.0.1), where duplicates were removed following Bramer’s method (Bramer et al., 2016).

### 2.2 Study selection and eligibility criteria

Titles and abstracts were screened by three reviewers independently using Rayyan (version 1.5.6) (Ouzzani et al., 2016). All microbiome studies published in English and conducted on individuals with PIs in any area of the body were included in this review. Animal, *ex vivo*, and *in vitro* studies, systematic reviews, case reports, editorials, commentaries, and conference abstracts were excluded. Studies were excluded if they focused solely on bacteraemia in PIs, skin infections, or bacterial treatment interventions. Furthermore, studies involving various chronic wound types (*e.g*. venous leg ulcers, diabetic foot ulcers, or other complex wounds) were excluded because of the absence of disaggregated data specific to PIs. The full texts were independently examined by two reviewers to generate a list of relevant studies. Discrepancies in study inclusion, also during title and abstract screening, were resolved through discussion with an experienced reviewer who participated in the final decision-making process.

### 2.3 Data extraction and methodological quality evaluation

Data extraction was conducted independently by two reviewers on a Excel template based on information concerning study identification (authors, publication year, journal, country), study characterisation (design, sample size, duration), population details (type, age, comorbidities), PI characteristics (grade, location), specific techniques used for microbiome identification (cultures, PCR, 16S rRNA sequencing, etc.), PI microbiome compositional analysis, interventional details (if applicable), and main findings for the studies.

The quality of each included study was evaluated using standardised risk-of-bias assessment tools appropriate for the study design. The National Heart, Lung, and Blood Institute (NHLBI) Quality Assessment Tool was used to assess study objectives, population selection, sample size justification, and outcome measurements (NHBLI, 2014). Each study was rated as having a low (75–100%), moderate (25–75%), or high (0–25%) risk of bias based on the proportion of criteria met within the tool.

## 3 Results

### 3.1 Literature search, study selection and study characteristics

**Figure 2:**
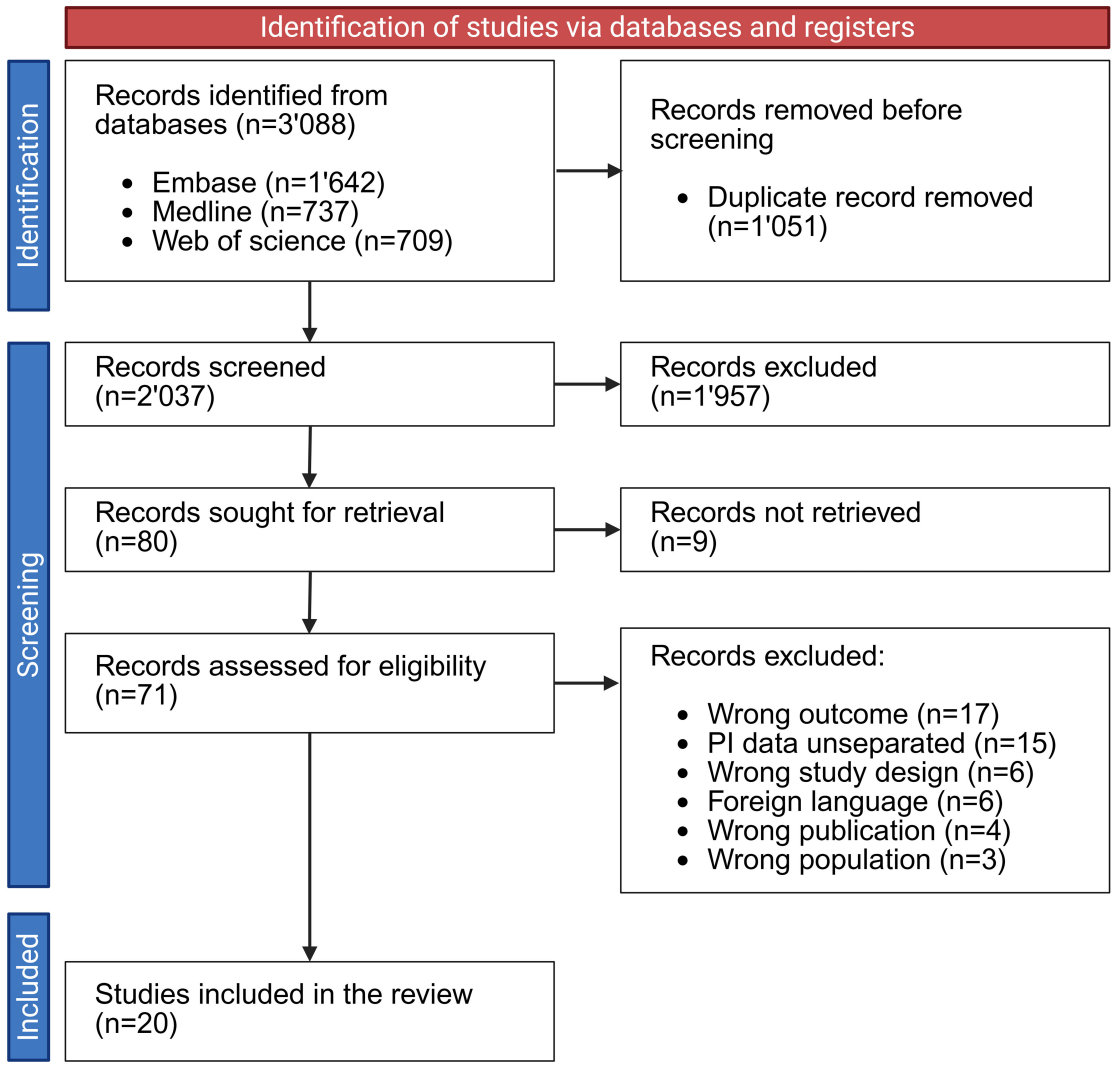
PRISMA flow diagram of the inclusion and screening and inclusion of studies focusing on the pressure injury microbiome [based on Page, 2021 (Page et al., 2021)]. Created in BioRender. Bertolo, A. (2025), https://BioRender.com/rxtpwrt.

The initial database search identified 3’088 records. After removing 1’051 duplicates, 2’037 titles and abstracts were screened for relevance (**Figure 2**). Seventy-one full-text articles were subsequently assessed, resulting in the inclusion of 20 studies in the final systematic review (**Tables 1**, **2**, and **Supplementary Table S1** in Supplemental Material File 2). Of the 20 studies, two were randomised controlled trials (Sopata et al., 2002; Sipponen et al., 2008) and one was an uncontrolled pre-post study (Goh et al., 2021). The other 17 studies were observational studies, including three case-control studies (Singh et al., 2015; de Wert et al., 2020; Lichtenthaler et al., 2023) and 14 cohort studies (Daltrey et al., 1981; Sapico et al., 1986; Brook, 1991; Andrianasolo et al., 2018; Sato et al., 2018; Fazel et al., 2019; Arisandi et al., 2020; Nagase et al., 2020; Dunyach-Remy et al., 2021; Shibata et al., 2021; Binsuwaidan et al., 2023; Kunimitsu et al., 2023; Wettstein et al., 2023; Yamashita et al., 2023). The quality of each study was examined using the NHLBI Quality Assessment Tool (**Supplementary Tables S2-S5**, in Supplemental material file 2). Among the studies included in this systematic review, only two showed a low risk of bias (Dunyach-Remy et al., 2021; Kunimitsu et al., 2023), and the remaining 18 studies demonstrated a moderate risk of bias.

**Table 1:** Summary of extracted data from studies included in this review using culture-based techniques for microbiome identification.

Authors	Type of study	Total patients (gender)	Population Investigated	Mean Age (y.o.)	Number of PIs	Outcomes measured related to PI microbiome	Results	Risk of bias
Andrianasolo et al., 2018	Retrospective cohort study	61 m;f= 46:15	SCI patients	47	64	Wound healing	***-*** Osteomyelitis was mostly polymicrobial (73%), with a predominance of *S. aureus* (47%), Enterobacteriaceae spp. (44%) and anaerobes (44%). ***-*** Treatment failure of PIs was associated to the presence of *Actinomyces* spp.-positive cultures at time of debridement.	Moderate
Arisandi et al., 2020	Prospective cohort study	20 m;f= 6:14	Hospitalized patients	88	20	PI recurrence rate	***-****Acinetobacter* spp. was significantly related to the development of recurrent PIs.	Moderate
Binsuwaidan et al., 2023	Retrospective cohort study	203 m;f= 186:17	SCI patients	NR	203	Wound healing, Antibiotic resistance	***-*** PIs were commonly infected by *Staphylococcus aureus, Pseudomonas aeruginosa*, and *Escherichia coli.* ***-*** Of the multidrug-resistant bacterial isolates, 26% were methicillin-resistant *S. aureus.*	Moderate
Brook et al., 1991	Retrospective cohort study	58 m;f= 31:27	Hospitalized patients	6	58	Wound healing	***-*** Although the flora found in children is similar to the one recovered in adults, *Haemophilus influenzae* is unique to this age group. ***-*** Anaerobic bacteria are frequently associated with PIs in paediatric patients, physicians should anticipate their presence for antimicrobial therapy	Moderate
Daltrey et al., 1981	Prospective cohort study	53 m;f= 11:42	Hospitalized patients	79	74	Wound healing	***-****Staphylococcus aureus, Proteus mirabilis, Pseudomonas aeruginosa, Bacteroides fragilis* and *Bacteroides asaccharolyticus* were found in infected PIs. ***-****P. mirabilis and P. aeruginosa* were associated with necrotic and enlarging PIs. ***-****Bacteroides* spp were associated with necrotic lesions. **o** The presence of *S. aureus* in a PI was not affecting the healing process.	Moderate
Fazel et al., 2019	Retrospective cohort study	55 m;f= 48:7	SCI patients	33	55	Wound healing, Antibiotic resistance	***-*** The most frequently isolated bacterium from smaller PIs at grade II was *Escherichia coli*, while in larger and advanced grade PIs was *Staphylococcus epidermidis*. ***-*** Sacral and trochanteric PIs were more commonly colonized with *E. coli*. ***-****Acinetobacter* and *Enterobacter* spp. were more prevalent in patients with poor bladder control ***-****E. coli* and *S. aureus* were antibiotic resistant.	Moderate
Goh et al., 2021	Pre-post study	16 m;f= 13:3	Hospitalized patients	66	16	Infection rate	**-** The most commonly isolated microorganisms were *Escherichia coli, Klebsiella pneumoniae, Enterococcus* species and *Candida* species, all of which were isolated in 31% of the patients. **+** Four sessions of UVC light therapy, with comparable effects to topical antiseptics used for wound cleansing, reduced the overall median CFU count of 79%. ***-*** Wounds with CFU counts >1000 CFU did not consistently respond to the UVC light therapy.	Moderate
Lichtenthaler et al., 2023	Case - control	120 m;f= 94:26	SCI patients	52	120	Wound healing	**o** Presence of a faecal diversion via a stoma is associated with minor changes of the microbial colonisation of perianal PIs. ***-****Streptococcus, Staphylococcus, Escherichia, Klebsiella, Pseudomonas, Proteus, Enterococcus* and *Candida* species were found in PIs. **o** The presence of a stoma does not affect PI healing time. **o** None of the species of the primary colonisation or secondary infection was associated with wound-healing disturbances or the length of the hospital stay.	Moderate
Sapico et al., 1986	Prospective cohort study	25 m;f= 21:4	SCI patients	34	25	Wound healing	***-****Streptococcus, Escherichia, Bacillus, Proteus* and *Enterococcus* were prominent in PIS presenting gross tissue necrosis. ***-*** In the absence of grossly necrotic tissue, *Staphylococcus*, and *Pseudomonas* became relatively more dominant in PIs. **o** No relationship was observed between the density of microorganisms and the eventual outcome of the myocutaneous flap procedure	Moderate
Sato et al., 2018	Retrospective cohort study	86 m;f= 59:27	Hospitalized patients	58	86	Granulation tissue formation	***-*** High bacterial burden suppressed granulation tissue formation in PIs. ***-****Corynebacterium* spp. and *S. aureus* were positively correlated with reduced cell proliferation in PIs, but that the quantity of *P. aeruginosa* was not.	Moderate
Singh et al., 2015	Case - control	25 m;f= 19:6	SCI patients	37	50	Infection rate	**+** Autologous platelet rich plasma (PRP) acted as an antimicrobial agent and reduced bacterial colonization of PIs from 92% to 24% of the patients following five weeks of application. **-***S. aureus* and *E. coli* were the most commonly cultured organisms.	Moderate
Sipponen et al., 2008	RCT	22 m;f= 9:13	Hospitalized patients	80 (74 control)	29	Wound healing	**+** Traditional resin salve is significantly more effective in the treatment of PIs than cellulose polymer gauzes. 92% of PIs healed in the resin group and 44% in the control group. **-***Staphylococcus aureus, Pseudomonas aeruginosa* and *Enterococcus faecalis* were the most common bacteria identified in PIs.	Moderate
Sopata et al., 2002	RCT	34 m;f= 16:18	Cancer patients	57	38	Wound healing	**-** Bacteriological assessment identified 92 species (80% were aerobic and 20% anaerobic), with the most common being *Staphylococcus* spp., *Enterococcus faecalis* and *Streptococcus pyogenes*. **o** Lyofoam/polyurethane foam dressing or Aquagel/hydrogel dressing were used to treat PIs. Efficacy, treatment times and **o** Healing rates were not different in the two groups. Neither the *Staphylococcus* spp. nor the anaerobes identified caused any clinical signs of infection or affected efficacy, treatment times and healing rates.	Moderate
Yamashita et al., 2023	Retrospective cohort study	40 m;f= 25:15	Hospitalized patients	59	69	PI complication rate, Antibiotic resistance	**-** Bacteria were detected in all PIs with early dehiscence, happening in 41% of the postsurgical cases. **o** MRSA was detected in 32%, but it was not found to be a risk factors of early wound dehiscence.	Moderate

Risk of bias evaluation for all studies was conducted with NIH tool. Results are identified as positive (+), uninfluential (o) and negative (-).

**Table 2:** Summary of extracted data from studies included in the systematic review using 16S rRNA techniques for microbiome identification.

Authors	Type of study	Total patients (gender)	Population Investigated	Mean Age (y.o.)	Number of PIs	Outcomes measured related to PI microbiome	Results	Risk of bias
Dunyach-Remy et al., 2021	Prospective cohort study	24 m;f= 15:9	SCI patients	62	24	Wound healing	**+***Corynebacterium* was positively correlated with wounds with an improved evolution and decreased in wounds with delayed healing. **+***Pelomonas* was only detected in wounds with “Improved” evolution. **-***Staphylococcus*, *Anaerococcus* and *Finegoldia* had high relative abundance in wounds that stagnated or worsened. **-***Proteus* and *Morganella* genera were only present in stagnated or worsened wounds. **-***Proteus, Morganella, Anaerococcus* and *Peptoniphilus* were associated within the same cluster in poor healing PIs.	Low
Kunimitsu et al., 2023	Prospective cohort study	18 m;f= 11:7	Hospitalized patients	86	22	Wound healing	**o** Predominant bacteria in wound and peri-wound skin were similar in the healing wounds. **-** In hard-to-heal PIs, the predominant were Bacteroides, Flavobacterium, *Anaerococcus*, Unidentified *Rikenellaceae*, and *Pseudomonas*.	Low
Nagase et al., 2020	Prospective cohort study	68 m;f= 28:40	Hospitalized patients	69	4	PI occurrence rate	**-** PI microbiomes with a high abundance of pathogens are formed from the adjacent skin microbiome.	Moderate
Shibata et al., 2021	Prospective cohort study	30 m;f= 13:17	Hospitalized patients	86	30	PI recurrence rate	**-** Lower skin hydration and a high rate of *Staphylococcus* species on the healed PI site were found in recurrent PIs.	Moderate
Wettstein et al., 2023	Prospective cohort study	27 m;f= 27:0	SCI patients	56	27	PI complication rate	**-** The cluster in PIs formed by *Staphylococcus, Streptococcus, Anaerococcus, Finegoldia, Peptoniphilus* and *Proteus* had higher rate of complications (67% rate) compared to the other clusters (~ 20% average rate). **-***Ezakiella* was more abundant in post-surgical complication patients’ PIs.	Moderate
de Wert et al., 2020	Case-control study	30 m;f= 18:12	Hospitalized patients	73	24	PI occurrence rate	**+** Presence of unclassified *Actinobaculum* and *Myobacterium vaccae* on skin were associated with absence of PIs. **-** The presence of unclassified *Enterococcus* has the highest impact on PI occurrence.	Moderate

Risk of bias evaluation for all studies was conducted with NIH tool. Results are identified as positive (+), uninfluential (o) and negative (-)

The three interventional studies included 72 patients and 83 PIs (Sopata et al., 2002; Sipponen et al., 2008; Goh et al., 2021). The remaining 17 studies were observational and included 918 patients with 901 PIs. Grade I PIs were not reported in any of the included studies. Instead, the focus was on more advanced grades: 8/20 studies included Grade II PIs (Sopata et al., 2002; Sipponen et al., 2008; Singh et al., 2015; Fazel et al., 2019; Arisandi et al., 2020; Goh et al., 2021; Shibata et al., 2021; Binsuwaidan et al., 2023), 11/20 included Grade III (Sopata et al., 2002; Sipponen et al., 2008; Singh et al., 2015; Fazel et al., 2019; Arisandi et al., 2020; Dunyach-Remy et al., 2021; Goh et al., 2021; Shibata et al., 2021; Binsuwaidan et al., 2023; Lichtenthaler et al., 2023; Wettstein et al., 2023), and 7/20 included Grade IV (Sipponen et al., 2008; Singh et al., 2015; Fazel et al., 2019; Dunyach-Remy et al., 2021; Binsuwaidan et al., 2023; Lichtenthaler et al., 2023; Wettstein et al., 2023). In nine studies, the PI grade was not clearly specified. PIs predominantly affected pressure-prone anatomical locations, including the sacrum (16/20) (Sapico et al., 1986; Sopata et al., 2002; Sipponen et al., 2008; Singh et al., 2015; Andrianasolo et al., 2018; Sato et al., 2018; Arisandi et al., 2020; de Wert et al., 2020; Nagase et al., 2020; Dunyach-Remy et al., 2021; Goh et al., 2021; Shibata et al., 2021; Binsuwaidan et al., 2023; Kunimitsu et al., 2023; Wettstein et al., 2023; Yamashita et al., 2023), ischium (9/20) (Sapico et al., 1986; Sipponen et al., 2008; Singh et al., 2015; Andrianasolo et al., 2018; Sato et al., 2018; Dunyach-Remy et al., 2021; Goh et al., 2021; Wettstein et al., 2023; Yamashita et al., 2023), trochanter (9/20) (Sapico et al., 1986; Sipponen et al., 2008; Singh et al., 2015; Sato et al., 2018; Arisandi et al., 2020; Dunyach-Remy et al., 2021; Shibata et al., 2021; Kunimitsu et al., 2023; Wettstein et al., 2023), and gluteal region (3/20) (Sopata et al., 2002; Goh et al., 2021; Binsuwaidan et al., 2023); in two studies, the location was not specified.

**Figure 3:**
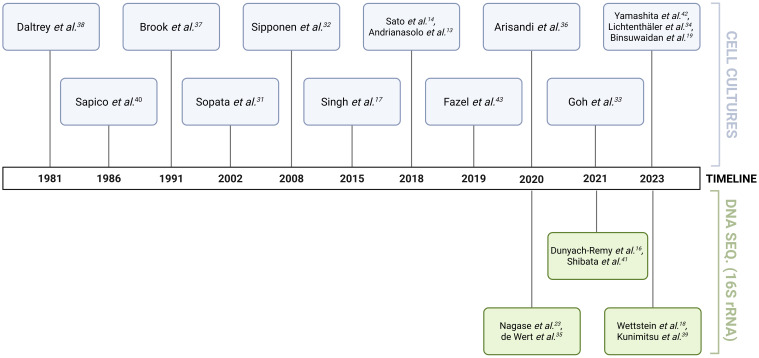
Timeline illustrating the methodological evolution of microbiome research on pressure injuries. The figure depicts the progressive inclusion and transition from conventional culture-based techniques (indicated in blue) to increasingly sophisticated molecular diagnostic methods (indicated in green). Created in BioRender. Bertolo, A. (2025), https://BioRender.com/8xqm4zt.

The study populations comprised individuals with SCI (8 studies; mean age 45 years) (Sapico et al., 1986; Singh et al., 2015; Andrianasolo et al., 2018; Fazel et al., 2019; Dunyach-Remy et al., 2021; Binsuwaidan et al., 2023; Lichtenthaler et al., 2023; Wettstein et al., 2023), hospitalised patients (11 studies; mean age 68 years) (Daltrey et al., 1981; Brook, 1991; Sipponen et al., 2008; Sato et al., 2018; Arisandi et al., 2020; de Wert et al., 2020; Nagase et al., 2020; Goh et al., 2021; Shibata et al., 2021; Kunimitsu et al., 2023; Yamashita et al., 2023), and cancer patients (1 study; mean age 59 years) (Sopata et al., 2002). Fifteen studies focused on adult patients (mean age 54 years), five studies focused on elderly patients (mean age 82 years) (Daltrey et al., 1981; Arisandi et al., 2020; Nagase et al., 2020; Shibata et al., 2021; Kunimitsu et al., 2023), and one study involved only paediatric patients (mean age 6 years) (Brook, 1991). Geographically, the studies were diverse, with six from Japan - the country with the world’s oldest population and therefore more exposed to the risk of PI development - eight from Europe, one from the USA, and the rest from other countries. The selected studies spanned more than four decades (1981–2023) (**Figure 3**). Notably, 11 of the included studies were published after 2020, of which six employed next-generation sequencing (NGS) technology for microbiome characterisation. The remaining studies relied on diverse, predominantly culture-based methods, many of which were not standardised, resulting in considerable methodological heterogeneity in the results. Given these inconsistencies, a qualitative and descriptive approach was adopted to examine the potential association between the PI microbiome and wound healing outcomes.

A comparative analysis of culture-based methods and molecular techniques, such as DNA sequencing, for microbiome characterisation in PIs, revealed significant discrepancies in diagnostic sensitivity. For example, while 23% of samples were reported as negative using traditional culture methods, DNA sequencing detected the presence of microbes in all specimens (Wolcott et al., 2010). Moreover, culture techniques identified only 27% of the genera present, frequently failing to detect anaerobic bacteria (Wettstein et al., 2023). The enhanced sensitivity provided by DNA sequencing was associated with improved clinical outcomes, including higher healing rates (48.5% in 2007 vs. 62.4% in 2009), reduced wound healing time (4 days vs. 5 days), and refined targeted antibiotic therapy (from 47% to 63%) (Wolcott et al., 2010). Molecular diagnostics also enable more sensitive determination of antibiotic susceptibility profiles. Notably, *S. aureus* exhibited frequent resistance to oxacillin due to the high prevalence of methicillin-resistant strains (MRSA), while *P. aeruginosa, E. coli*, and *K. pneumoniae* demonstrated variable susceptibility patterns (**Table 3**) (Sipponen et al., 2008; Wolcott et al., 2010; Fazel et al., 2019; Binsuwaidan et al., 2023).

**Table 3:** Antibiotic susceptibility and resistance in bacteria resident in PIs.

Bacterial species	Antibiotic susceptibility	Antibiotic resistance	Publications
*Escherichia* spp.	Cephalosporins, gentamicin, imipenem, piperacillin-tazobactam, trimethoprim	Ampicillin, ciprofloxacin, trimethoprim-sulfamethoxazole	Binsuwaidan et al., 2023; Fazel et al., 2019; Wolcott et al., 2010
*Klebsiella* spp.	Aminoglycosides, cefepime	n.a.	Binsuwaidan et al., 2023
*Staphylococcus* spp.	Clindamycin, linezolid, mupirocin, trimethoprim	Methicillin, oxacillin, penicillin, trimethoprim-sulfamethoxazole	Binsuwaidan et al., 2023; Fazel et al., 2019; Sipponen et al., 2008
*Pseudomonas* spp.	Ceftazidime, ciprofloxacin, gentamicin, meropenem	n.a.	Binsuwaidan et al., 2023; Wolcott et al., 2010

### 3.2 Characterization of PI microbiome

**Figure 4:**
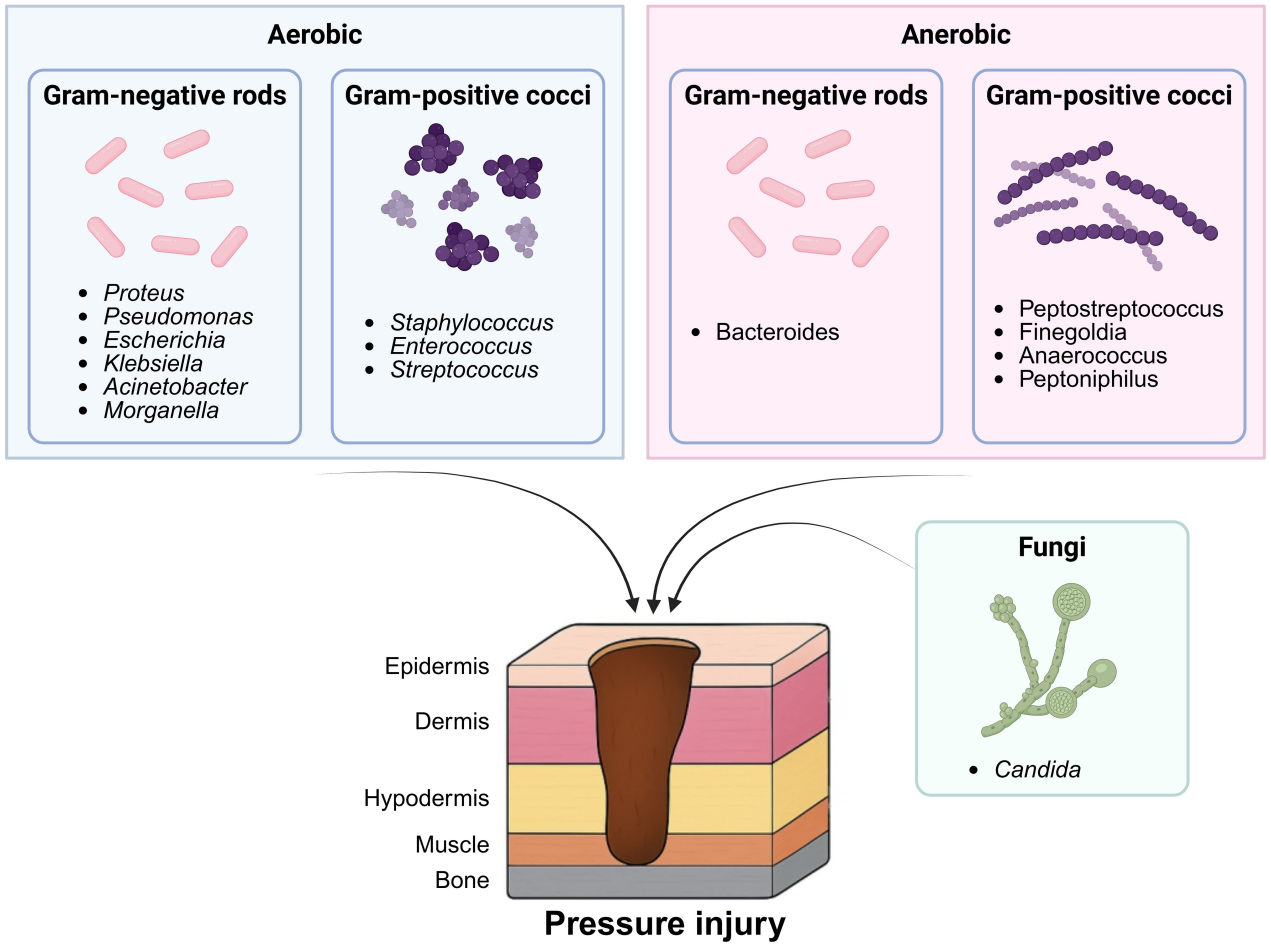
Microbial diversity associated with pressure injuries. This diagram illustrates the common microorganisms isolated from PIs, categorised by oxygen requirement, gram staining, and morphology. These microbial populations colonise different tissue layers of PIs and may influence the wound healing process. Created in BioRender. Bertolo, A. (2025), https://BioRender.com/yshcx88.

Bacterial diversity within the PI microbiota plays a crucial role in colonisation, infection, and wound chronicity (Bar et al., 2021). *Firmicutes, Bacteroidetes, Actinobacteria*, and *Proteobacteria* were identified as dominant phyla in PIs, with *Staphylococcus aureus*, *Pseudomonas aeruginosa*, *Escherichia coli*, *Klebsiella pneumoniae*, *Enterococcus* spp. and *Proteus mirabilis* reported as the most prevalent species (**Figure 4**) The presence of these common pathogenic bacteria, especially in large quantities, significantly exacerbates wound chronicity, impairs fundamental healing processes, and increases the risk of severe local and systemic infections (**Supplementary Table S6**, in Supplemental material file 3). *S. aureus* prevalence ranged from 10%–90% and it was detected in all studies. In the two RCTs, *S. aureus* was detected in 20.7% (Sopata et al., 2002) and 33% (Sipponen et al., 2008) of baseline PI samples, respectively. *P. aeruginosa* was found in 4%-80% of PIs by three interventional, two case-control and eleven cohort studies (Daltrey et al., 1981; Sapico et al., 1986; Brook, 1991; Sopata et al., 2002; Sipponen et al., 2008; Singh et al., 2015; Andrianasolo et al., 2018; Sato et al., 2018; Fazel et al., 2019; Arisandi et al., 2020; Dunyach-Remy et al., 2021; Goh et al., 2021; Binsuwaidan et al., 2023; Kunimitsu et al., 2023; Lichtenthaler et al., 2023; Wettstein et al., 2023). In two prospective cohort studies involving 53 and 25 participants*, P. aeruginosa* presence was linked to non-healing wounds, persisting in non-necrotic tissues (Daltrey et al., 1981; Sapico et al., 1986).

The prevalence of gram-negative *Enterobacteriaceae* varied widely: *Escherichia coli* 6%-56% (Daltrey et al., 1981; Brook, 1991; Wolcott et al., 2010; Fazel et al., 2019; Goh et al., 2021; Lichtenthaler et al., 2023), *Proteus mirabilis* 1%-57% (Daltrey et al., 1981; Brook, 1991; Sopata et al., 2002; Wolcott et al., 2010; Sato et al., 2018; Fazel et al., 2019; Goh et al., 2021; Binsuwaidan et al., 2023; Lichtenthaler et al., 2023; Wettstein et al., 2023), and *Klebsiella* spp. 1%-31% (Daltrey et al., 1981; Wolcott et al., 2010; Sato et al., 2018; Nagase et al., 2020; Goh et al., 2021; Lichtenthaler et al., 2023). Four cohort studies have linked gram-negative bacteria to biofilm formation thus delaying healing, such as *Escherichia coli* and *Enterobacter* spp (Dunyach-Remy et al., 2021).*, Proteus mirabilis* and *Bacteroides* spp (Daltrey et al., 1981; Sapico et al., 1986; Dunyach-Remy et al., 2021)., and anaerobes that dominate necrotic wounds (such as *Bacteroides fragilis* and *Peptostreptococcus* spp (Sapico et al., 1986; Andrianasolo et al., 2018; Dunyach-Remy et al., 2021).). *Proteus mirabilis* was frequently associated with chronic, non-healing, or necrotic wounds, but at a moderate prevalence (Sopata et al., 2002; Binsuwaidan et al., 2023). *Candida* spp. can be found within PIs and are considered opportunistic pathogens, as observed in one interventional and one observational study (Andrianasolo et al., 2018; Goh et al., 2021).

### 3.3 Microbiome variations based on patient populations and PI characteristics

Microbiome variations in different clinical settings were highlighted in a prospective cohort study comparing 19 healthy young adults, 18 ambulatory older adults, and 31 bedridden older inpatients. The bedridden group exhibited higher levels of *Escherichia–Shigella*, *Bifidobacterium*, *Bacteroides*, *Enterococcus*, *Brevibacterium*, and *Klebsiella* than healthy and ambulatory individuals. Alpha diversity was significantly higher, and microbiome clustering was distinct in bedridden patients, with no major shifts in microbiome composition before and after symptom onset (Nagase et al., 2020). In geriatric patients, a prospective cohort study with 53 participants found that gram-negative rods (such as *E. coli, K. pneumoniae, P. mirabilis, and Enterobacter* spp.) were present in 71% of necrotic or stagnant wounds but only in 9% of healing wounds. *P. mirabilis* and *P. aeruginosa* correlated with necrosis and enlarging lesions, while *S. aureus* showed no clear association with healing trends (Daltrey et al., 1981). In a retrospective cohort study of 58 paediatric patients, using culture-based methods, *S. aureus* was the most prevalent species (43%), often mixed with *Peptostreptococcus* spp. and *Bacteroides* spp. Gram-negative aerobes, such as *P. aeruginosa* and *E. coli*, were less frequent, and wounds harboured an average of 2.3 bacterial species per wound, including the bacteria *Haemophilus influenzae* (Brook, 1991). In individuals with SCI, *S. aureus* was the most frequently identified bacterium across two case-control studies (25–120 participants) and four cohort studies (23–203 participants), consistently reported as one of the most common isolates, along with *Pseudomonas* and *Proteus.* Other commonly found bacteria included *Enterococcus* spp. and *K. pneumoniae*, often acquired from enteric flora (Singh et al., 2015; Fazel et al., 2019; Dunyach-Remy et al., 2021; Binsuwaidan et al., 2023; Lichtenthaler et al., 2023; Wettstein et al., 2023).

Four cohort studies have explored the relationship between microbiome composition, wound location, PI severity, and morphology. In one study involving 55 participants with SCI, *E. coli* was significantly associated with smaller wound sizes and sacral/trochanteric PIs, whereas *Klebsiella* spp. showed a strong link to sacral PIs (Fazel et al., 2019). Another study of 203 patients with SCI found that *S. aureus* was the most prevalent in sacral (28%) wounds, followed by *P. mirabilis* (21%) and *P. aeruginosa* (16%) (Binsuwaidan et al., 2023). Two additional cohort studies using NGS reported higher alpha diversity in grade IV than in grade III PIs, and the PI microbiome was compositionally similar to the surrounding skin, implying that the skin microbiome was propagated into the injury (Nagase et al., 2020; Wettstein et al., 2023). In a cohort study involving 30 patients using NGS, the microbial profiles of unaffected skin were compared to those of PIs. *Enterococcus* abundance was positively associated with PI occurrence, and a subset of four species (*Enterococcus, Allobaculum, Eubacterium*, and *Staphylococcus*) correctly classified PI occurrence in 74% of patients (de Wert et al., 2020). Deep wounds exhibited greater diversity, with bacteria such as *Bacteroides, Flavobacterium, Anaerococcus*, and *Pseudomonas* (Kunimitsu et al., 2023). A microbial cluster dominated by *Staphylococcus, Streptococcus, Anaerococcus, Finegoldia, Peptoniphilus*, and *Proteus* was associated with a higher complication rate (67% average rate) than the other clusters (20% average rate), as reported in a prospective cohort study of 27 participants (Wettstein et al., 2023). Risk factors for early dehiscence (40% incidence) included elevated C-reactive protein (CRP) levels, low albumin levels, use of musculocutaneous flaps for defect reconstruction, and longer duration of surgery; however, no significant associations with bacterial species or MRSA were observed. Surgical site infections were the primary cause of early dehiscence (Yamashita et al., 2023).

### 3.4 PI microbiome associations with healing outcomes

**Figure 5:**
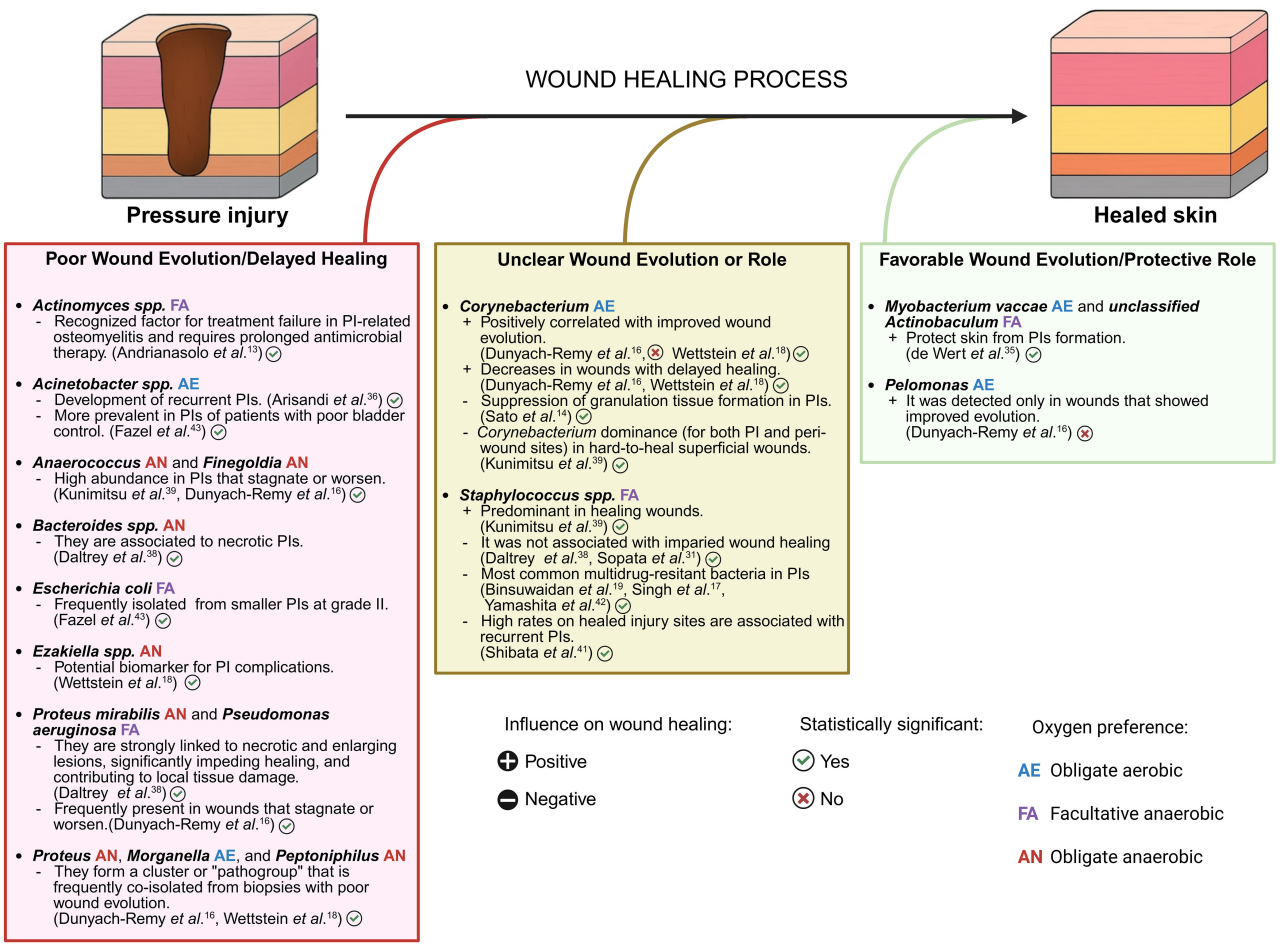
Association of different bacteria with the wound healing process in pressure injuries. This diagram illustrates the bacteria present in PIs and their negative (left), ambiguous (middle), and positive (right) effects on wound healing. The mechanism of action and statistical significance of the findings for each bacterium are described. Created in BioRender. Bertolo, A. (2025), https://BioRender.com/axckqnn.

Wound healing outcomes have been associated to specific bacteria present in PI microbiome (**Figure 5**). Some bacterial genera were associated with favourable wound evolution. *Corynebacterium* was positively correlated with improved wound evolution and decreased wounds with delayed healing (Dunyach-Remy et al., 2021; Wettstein et al., 2023). However, a cohort study comparing 22 wound and peri-wound microbiomes in superficial and deep PIs found that *Corynebacterium* was the predominant bacterium in hard-to-heal PIs and in the presence of *S. aureus*, it impaired cell proliferation in granulation tissue, challenging its real contribution to improved wound healing (Sato et al., 2018; Kunimitsu et al., 2023). The genera *Myobacterium vaccae* and *unclassified Actinobaculum* were associated with the absence of PIs (de Wert et al., 2020). The genus *Pelomonas* was only detected in wounds with “Improved” evolution, but the difference was not statistically significant (Dunyach-Remy et al., 2021). Conversely, other bacterial species were strongly associated with poor wound healing. A 28-day follow-up cohort study examining the evolution of the microbiome in 24 PIs found that *P. mirabilis* and *P. aeruginosa* were associated with necrotic and enlarging lesions, leading to significantly lower healing rates (Daltrey et al., 1981; Dunyach-Remy et al., 2021; Wettstein et al., 2023). These genera, along with *Morganella*, were found in wounds that stagnated or worsened (Dunyach-Remy et al., 2021). Strict anaerobic bacteria, particularly *Anaerococcus* and *Finegoldia*, were found in higher abundance in wounds that stagnated or worsened, and their presence was associated with lower healing rates (Daltrey et al., 1981; Dunyach-Remy et al., 2021; Wettstein et al., 2023). These anaerobes often form symbiotic relationships within polymicrobial biofilms, creating an environment conducive to their persistence and contributing to non-healing PIs. *Actinomyces* spp. infection was a known determinant of treatment failure, often necessitating prolonged antimicrobial therapy of at least six months (Andrianasolo et al., 2018). PI recurrence (14% of cases) after conservative treatment within two months was linked to elevated pH, *Acinetobacter* spp., and higher interface pressure, as reported in a cohort study of 20 participants (Arisandi et al., 2020). While *S. aureus* was a common isolate in PIs, and some studies did not find a correlation between its presence and healing trends (Daltrey et al., 1981; Sopata et al., 2002), higher rates of this bacteria on healed sites were associated with recurrent PIs (Shibata et al., 2021). In a prospective cohort study including 18 hospitalized patients, the rate of the microbial similarity between deep PIs and periwound skin was lower in hard-to-heal wounds than in healing wounds (Kunimitsu et al., 2023).

An investigation of factors impairing cell proliferation during granulation tissue formation in a cohort study involving 86 PIs found a significant negative correlation between low cell proliferation rates and high bacterial counts, demonstrating that bacterial count was a significant predictor of poor cell proliferation (Sato et al., 2018). A RCT with 61 participants identified a critical bacterial threshold for infection, suggesting that ≤10^5^ CFU/g of tissue is essential for normal wound healing. Among the PIs analysed, 96% had <10_2_ CFU/g, 3% had 10_2_ to 10_5_ CFU/g, and only 1% exceeded 10^5^ CFU/g. In the latter group, some cases exceeding the 10^5^ CFU/g threshold returned to 10^5^ or fewer CFU/g in the subsequent week without specific treatment, with *Bacillus* or *Corynebacterium* present (Robson et al., 1999). However, the mere presence of bacteria can retard wound healing, even if the count is less than 10^5^ CFU/g (Sato et al., 2018). A cohort study, counting bacterial loads in deep and necrotic PIs of 25 patients with SCI, found 5.8 isolates/patient with a density of 6.4 log_10_/g and similar counts for aerobes and anaerobes (Sapico et al., 1986). In contrast, in deep PIs without necrosis and as PIs healed, 1.5 aerobic and 0.2 anaerobic isolates/patient were found, with mean densities of 2.7 and 0.1 log_10_/g, respectively.

### 3.5 The association between different treatment modalities and PI microbiome

Of the 20 studies reviewed, five focused on surgical outcomes (Sapico et al., 1986; Wolcott et al., 2010; Andrianasolo et al., 2018; Sato et al., 2018; Wettstein et al., 2023), and six examined conservative approaches for PIs (Sopata et al., 2002; Sipponen et al., 2008; Singh et al., 2015; Arisandi et al., 2020; Dunyach-Remy et al., 2021; Goh et al., 2021). The remaining studies did not specify treatment modalities and primarily addressed other aspects of the PI microbiome (Daltrey et al., 1981; Brook, 1991; Fazel et al., 2019; de Wert et al., 2020; Nagase et al., 2020; Shibata et al., 2021; Binsuwaidan et al., 2023; Kunimitsu et al., 2023; Lichtenthaler et al., 2023). Most studies have not directly investigated the association of PI treatment and wound microbiome. However, several studies investigating conservative interventions have reported notable effects on both wound healing and microbiome composition. One RCT compared the use of a resin salve to conventional sodium carboxymethylcellulose hydrocolloid polymer dressings in 13 participants with PIs and 9 controls (Sipponen et al., 2008). The resin salve demonstrated superior clinical efficacy, as evidenced by higher rates of complete healing, reduced ulcer width and depth, and enhanced antimicrobial effects. The second RCT compared the efficacy, healing rates, and treatment duration of Lyofoam and Aquagel dressings on PIs in 18 and 20 cancer patients, respectively (Sopata et al., 2002). No statistically significant differences were found between the groups in terms of healing rates or treatment duration, and none of the identified bacterial species influenced the clinical outcomes. In a pre-post study involving 16 patients, ultraviolet-C therapy resulted in a 79% reduction in bacterial load (when colony-forming unit levels were <500), with complete bacterial clearance observed in 29% of the cases (Goh et al., 2021). Similarly, a case-control study involving 25 patients and 24 controls found that Platelet-Rich Plasma (PRP) therapy significantly reduced bacterial colonisation in stage IV PIs from 92% to 24%, whereas the saline-treated control group showed only a modest decrease, from 84% to 76% (Singh et al., 2015).

## 4 Discussion

In this systematic review, we found that the predominance of anaerobes in the PI microbiome negatively affects wound healing time, with bacterial infections being one of the main reasons for delayed wound healing, which leads to prolonged hospitalisation and increased healthcare costs, both in conservative and surgical management.

### 4.1 The microbiome of PIs

The most predominant species identified in PIs were *S. aureus* and *P. aeruginosa* and Gram–negative *Enterobacteriaceae* (*E. coli*, *Enterobacteriaceae* spp., *K. pneumonia* and *P. mirabilis*). *S. aureus* is consistently identified as one of the most, if not the most, prevalent or commonly isolated organisms in PIs, often appearing in nearly half of affected wounds or as the primary bacteria. Anaerobes were most common in necrotic tissue and linked to foul odour, although anaerobes were also found in six odourless cases, with *P. aeruginosa* and *S. aureus* prevailing as necrosis resolved (Sapico et al., 1986). The facultative nature of *P. aeruginosa* is advantageous for survival in low oxygen levels in non-healing wounds, dominating other aerobic species under the same conditions.

The bacterial groups commonly detected in PIs originate primarily from the local environment, including the contiguous skin, gastrointestinal tract, and urogenital mucosa (Gomes et al., 2022; Wettstein et al., 2023). The predominant organisms in PIs vary significantly depending on the location (Fazel et al., 2019; Binsuwaidan et al., 2023) and severity grade of the PIs (Fazel et al., 2019; Wettstein et al., 2023). Different body areas (*e.g.* sacral, gluteal, and trochanteric) are exposed to varying levels of moisture, pressure, and contamination (*e.g.* proximity to the perineal area), which can favour specific bacteria. For instance, *E. coli* and *Klebsiella* spp. were significantly associated with larger wounds and two PI locations (sacral and trochanteric) (Fazel et al., 2019), suggesting a potential role for these bacteria in worsening or maintaining PI severity. The strong association of *S. aureus* with gluteal and sacral wounds and the prevalence of *P. mirabilis* and *P. aeruginosa* (Binsuwaidan et al., 2023) suggest that the microbiome composition may differ across PI locations and may directly or indirectly influence healing by altering inflammation, tissue integrity, or resistance to treatment. Some bacteria, such as *P. aeruginosa* and *S. aureus*, form biofilms that protect microbes from immune responses and antibiotics and can trigger different immune responses, leading to chronic inflammation and delayed healing.

Higher alpha diversity in grade IV PIs than in grade III PIs, and greater diversity in PIs than in the surrounding skin indicate that more severe wounds may harbour a more diverse microbial community (Wettstein et al., 2023). This diversity may reflect a more complex microbial environment that is associated to healing. A diverse microbiota may lead to increased microbial competition, potentially disrupting the balance between beneficial and harmful organisms. This can create a less stable environment that hinders the healing process. It may also trigger multiple immune pathways, increasing chronic inflammation, impairing tissue repair, and prolonging the inflammatory phase of healing. Severe wounds may allow colonisation by opportunistic and antibiotic-resistant bacteria, complicating treatment and delaying wound closure in both conservative and surgical treatments. In surgical sites, *Ezakiella* has been identified in wounds with post-surgical complications, suggesting its potential involvement in PI-related complications (Wettstein et al., 2023). Although some bacteria (for example*, S. aureus* and *P. aeruginosa*) can be found in the microbiomes of PIs in both paediatric and geriatric populations, the presence of *Haemophilus influenzae*, although measured by a culture-based method, was unique to the paediatric age group (Brook, 1991). Age is usually considered a risk factor for recurrent PIs in older patients, but the results could not establish a statistically significant association (Arisandi et al., 2020; Shibata et al., 2021).

Interestingly, all studies focused primarily on bacteria, whereas fungi and viruses were rarely mentioned or entirely omitted. The main fungal species identified in PIs was *Candida*, although its presence was noted only by appearance, without any analysis of its functional role in wound healing. Viruses were not mentioned, despite the reasonable assumption that they could influence wound pathology. This is partly due to the use of the 16S rRNA sequencing methodology, which detects only bacterial DNA. Although some culture-based methods have been used, they primarily target bacteria and may lack the sensitivity to effectively identify fungi or viruses.

### 4.2 Microbial detection methods for PIs

A significant advancement achieved in the last decade is the improvement of bacterial identification using molecular methods, compared to traditional culture methods. All bacteria were identified using culture-based or molecular techniques (DNA analysis). Modern molecular techniques, such as PCR and NGS, have expanded the list of identified species linked to deep PIs, particularly anaerobes that were previously difficult to culture, such as *Dietzia, Paracoccus, Nosophingobium, Delftia*, and *Pelomonas* (Dunyach-Remy et al., 2021). DNA sequencing allowed for the reliable detection of often missing anaerobes, such as *Finegoldia, Anaerococcus, Peptoniphilus*, and *Peptostreptococcus*, and underestimated species, such as *Pseudomonas, Streptococcus*, and *Bacteroides*. However, cultures reportedly overestimated *Enterococcus* and *Acinetobacter* compared to DNA sequencing (Wettstein et al., 2023).

Molecular pathogen diagnostics have been shown to significantly shorten healing times for PIs due to faster and a more precise prescription for the use of antibiotics especially when associated with antibiogram. For example, the implementation of molecular diagnostics at one wound care centre led to a 12-days (23%) decrease in healing time for PIs (Wolcott et al., 2010). These advanced diagnostics are considered more reliable than traditional cultures, which often fail to identify all microbial species and biofilm phenotypes due to different techniques being used and is inherently susceptible to human error. The need for a more standard method for a universal and accessible use in different hospital settings, will allow for the enhanced detection and targeted antibiotic therapies, biofilm-based and local wound care, and reduces the use of broad-spectrum antibiotics - even against resistant strains, such as MRSA. However, not all detected DNA reflects active infection because some may be from dead or transient microbes. Therefore, the findings must be interpreted carefully, ideally in combination with clinical data and traditional cultures, to distinguish true pathogens from false-positive results. In summary, molecular tools are transforming wound microbiology by confirming known pathogens and exposing a more complex and dynamic microbial landscape in PIs.

### 4.3 Wound healing and the microbiome of PIs

The impact of infection on PI recovery time is substantial, with bacterial infections being a relevant reason for delayed wound healing in chronic wounds (Edwards and Harding, 2004). High bacterial loads and biofilm formation can amplify wound inflammation and significantly delay healing by impairing various processes in the wound healing cascade (Malone et al., 2017). Traditionally, a bacterial load exceeding 10^5^ CFU per gram of tissue is considered detrimental to healing, although β-haemolytic *Streptococci* can disrupt healing at even lower concentrations because of their unique virulence factors (production of fibrinolysins, leukocidins, and hemolysins) (Robson et al., 1999). Both surgical and spontaneous wound healing processes are negatively affected by elevated microbial loads (Cogen et al., 2008).

In healthy individuals, commensal microbiota typically exhibits non-pathogenic behaviour and may protect against harmful microbial colonisation. For instance, *S. epidermidis* can inhibit nasal colonisation by *S. aureus* (Otto et al., 2001). However, disruption of epithelial integrity allows commensal organisms to contribute to delayed healing or infection, depending on their type and burden (Iwase et al., 2010). Furthermore *Corynebacterium* has been associated with favourable wound evolution and reduced delayed healing, however, it was also found to be predominant in hard-to-heal wounds, and its co-presence with *S. aureus* appeared to impair granulation tissue cell proliferation (Sato et al., 2018; Kunimitsu et al., 2023). This suggests that its role may be context-dependent or influenced by microbial interaction. Only two bacteria, *Mycobacterium vaccae* and unclassified *Actinobaculum*, were associated with the absence of PIs, so we can speculate on a protective role in maintaining skin integrity (de Wert et al., 2020). In contrast, several genera were consistently associated with poor healing trajectories. *P. mirabilis* and *P. aeruginosa* along with *Morganella*, and *Actinomyces* spp. were prevalent in stagnant or deteriorating wounds, suggesting a potential role in driving or sustaining tissue damage (Daltrey et al., 1981; Dunyach-Remy et al., 2021; Wettstein et al., 2023). In addition to specific pathogens, dysbiotic wound microbiota, characterised by an imbalance in commensal flora, can lead to inflammation and delayed healing (Nagase et al., 2020; Kunimitsu et al., 2023).

Across both SCI and elderly bedridden populations, PI microbiota was predominantly influenced by enteric flora due to proximity to the anus and frequent incontinence. Wound contamination from faeces and urine, involving bacteria such as *E. coli*, *Enterococcus* spp., *K. pneumoniae*, *Bifidobacterium* spp., and *Bacteroides* spp., leads to increased colonisation and a slower healing rate in PIs (Brook, 1991; Singh et al., 2015; Nagase et al., 2020). A higher abundance of gut-related bacteria is also observed in the skin and wounds of bedridden patients, which can lead to wound infection and potentially slow healing (de Wert et al., 2020; Nagase et al., 2020; Wettstein et al., 2023). The survival and proliferation of these gastrointestinal organisms in the PIs are favoured by the loss of skin barrier integrity and changes in the local skin microenvironment (Nagase et al., 2020). However, SCI and elderly bedridden populations differ in microbial environment, microbial burden and predictors of healing (**Supplementary Table S7**, in Supplemental material file 3). Although both groups develop PIs over the sacral, ischial, and trochanteric regions, SCI patients commonly show higher rates of gram-negative perineal colonization associated with neurogenic bladder dysfunction (Dana and Bauman, 2015; Fazel et al., 2019), whereas elderly patients exhibit profound skin dysbiosis, diminished commensal populations, and increased abundance of gut-associated bacteria (Nagase et al., 2020; Wettstein et al., 2023). Microbial burden is substantial in both groups, but SCI lesions, particularly those with tissue necrosis, demonstrate consistently high deep-tissue bacterial loads (Sapico et al., 1986), while geriatric lesions show a broader range of superficial contamination levels and a lower threshold for defining infection (Daltrey et al., 1981; Robson et al., 1999). In the elderly, recurrence of a PI after conservative management is strongly associated with the presence of *Acinetobacter* spp. and persistently high post-healing skin pH (Arisandi et al., 2020). In contrast, in individuals with SCI, delayed or worsening wounds are associated with anaerobic genera such as *Anaerococcus* and *Finegoldia* at baseline, and later with *Proteus* and *Morganella*, while treatment failure in deep surgical infections (including osteomyelitis) is linked to *Actinomyces* spp (Andrianasolo et al., 2018; Dunyach-Remy et al., 2021; Wettstein et al., 2023).

Effective conservative management of the PI microbiome through strategies such as targeted topical antimicrobials, silver sulfadiazine, and platelet-rich plasma therapy have demonstrated significant improvements in healing times (Wolcott et al., 2010; Singh et al., 2015; Chen et al., 2023). Surgical reconstruction is performed, especially for complex and deep PIs or when local wound care is insufficient (Andrianasolo et al., 2018). Although surgical flap reconstructions are widely used, the specific role of the microbiome in surgical wound healing remains poorly defined. Interestingly, a clinical trial analysing 20 wounds before and after debridement observed no major changes in microbiome diversity or community structure, with dominant bacterial taxa remaining consistent throughout the process (Verbanic et al., 2020). Postoperative complications, including surgical site infections and early wound dehiscence, are common and often require additional surgical interventions (Andrianasolo et al., 2018; Wettstein et al., 2023; Yamashita et al., 2023). Additional risk predictors for major complications identified in a recent study include reduced glomerular filtration rate and deficiencies in vitamin D, vitamin B12, and sodium (Fahndrich et al., 2025). Also the high rate of recurrence (approximately one in four treated PIs) further highlights persistent challenges (Dunyach-Remy et al., 2021). Risk factors for recurrence include elevated skin pH, the presence of *Acinetobacter* spp., and higher interface pressure on the healed wound site (Arisandi et al., 2020). This may be due to *Acinetobacter* spp. ability to form biofilms on the skin surface and invade deeper tissues, potentially impairing the skin barrier (Arisandi et al., 2020). For deep PIs, healing wounds tend to exhibit similar bacterial compositions in both the wound and peri-wound skin, whereas hard-to-heal wounds show distinct microbial compositions, underscoring the importance of this similarity for effective healing (Kunimitsu et al., 2023). The failure to heal may be linked to a microbial community within the PIs that deviates significantly from the commensal skin flora.

### 4.4 Colonization versus ‘infection’- still a challenged definition today

Due to skin breakdown, chronic wounds such as PIs are colonised by bacteria that originate from the external environment, periwound skin, or gastrointestinal flora. A better understanding of bacterial colonisation and wound infection is required to better manage therapeutic interventions that reduce morbidity and mortality (Dana and Bauman, 2015).

Despite extensive research, no universally accepted definitions of “infection” and “colonisation” of PIs exist. Bendy et al. (1964) proposed to set the threshold equal to bacterial loads exceeding 10^6^/ml of gram-negative bacilli and coagulase-positive *Staphylococcus*, at which bacteria produce harmful enzymes and toxins that damage tissue (Bendy et al., 1964). Their RCT demonstrated that suppressing bacteria using topical gentamicin significantly improved the wound healing. However, a controversial result shows that *S. aureus* can be present in concentrations ranging from 1x10² to 9x10^8^/g tissue without clinical signs of infection, and without the use of antibiotics, with no relation to wound grade or dressing (Sopata et al., 2002). The presence of high concentrations of *S. aureus* without clear signs of infection challenges the traditional definition of an infection. This suggests that *S. aureus* may not always act as a pathogen in PIs, particularly when it coexists with the host without triggering an inflammatory response. Such cases may reflect bacterial adaptation, host tolerance, or a complex host-microbe equilibrium that prevents tissue damage. Moreover, antibiotic resistance in *S. aureus* does not necessarily correspond to pathogenic activity if the bacterium does not cause clinical symptoms. Other bacterial species may exhibit similar behaviour, with the transition from colonisation to infection depending not only on bacterial load but also on host factors, microbial virulence, and the wound environment. The commonly accepted threshold of 10^5^ CFU/g as the marker of transition from colonisation to infection may therefore be an oversimplification, as microbial pathogenicity appears to be more species-specific and context-dependent than previously thought (Robson et al., 1999). However, extrinsic and intrinsic factors may have an equally meaningful impact on wound healing. Wysocki et al. (2002) emphasised the importance of optimal wound care in preventing the progression from colonisation to infection, considering it a cornerstone of good clinical practice (Wysocki, 2002).

### 4.5 Limitations of the systematic review

While many studies have highlighted a strong association between specific bacteria and delayed healing, some studies have presented contrasting findings. A study on 25 PIs found no consistent relationship between quantitative tissue bacterial counts and the outcome of myocutaneous rotation flap surgery (Sapico et al., 1986). Another study on superficial PIs in patients with advanced cancer found that variations in the number and types of bacteria did not correlate with the grade of ulcer or dressing used, and none of the 92 isolated species had a more adverse effect on healing than the others (Sopata et al., 2002). One study found that microbial colonisation (including specific species or multidrug-resistant germs) was not associated with wound-healing disturbances or hospital stay length in anus-near PIs, even after faecal diversion (Lichtenthaler et al., 2023).

In addition, the available evidence is largely of moderate risk of bias, with 85% of the studies derived from observational studies, which limits the ability to draw definitive conclusions from the results. The small sample sizes, variability in statistical methods (including inconsistent adjustments in p-values), and differing ways of reporting results further limit the generalisability of the findings. Importantly, this review is limited by considerable methodological heterogeneity across studies spanning 1981–2023, with many relying on non-standardised, culture-based methods, and only a minority using NGS technologies.

### 4.6 Future outlook

Future research must address the critical challenge of clearly distinguishing between infection and colonisation in PIs, to improve our understanding of species pathogenicity in local wound environments. Many studies using NGS techniques have focused on the differences between PI microbiomes and peri-wound areas or other body sites. This approach may help answer key questions, such as the most influential source of diversity in PI microbiomes (whether derived from the patient’s microbiome or the surrounding environment) and the pathogenic effects of bacteria. A main factor influencing pathogenicity is the host’s immune response to microbes (Brodsky and Medzhitov, 2009). Each patient possesses a unique microbiome that interacts differently with the environment, thereby affecting the immune system and health outcomes. Additionally, antibiotic use in PI therapy may have limited efficacy in chronic wounds, as they may not reach the targeted tissue levels or when bacteria persist in antibiotic-resistant biofilms (Smith et al., 2020). Consequently, these therapies tend to have minimal impact on microbiome diversity (Robson et al., 1999; Wolcott et al., 2010; Dunyach-Remy et al., 2021; Goh et al., 2021; Wettstein et al., 2023). While systemic antibiotics are often used for severe infections such as osteomyelitis following surgical debridement and flap coverage (Andrianasolo et al., 2018), cautious selection is required because of the high risk of MDR infections (Filius and Gyssens, 2002). Topical or narrow-spectrum treatments are recommended for localised infections (Bowler et al., 2001). Addressing these questions could facilitate more targeted interventions to prevent the spread of pathogenic species and improve healing outcomes in the future.

## 5 Conclusions

In conclusion, this review underscores the complex association between PI microbiome and wound healing. The interplay between dominant bacterial phyla, biofilm formation, and microbial diversity directly and indirectly modulates inflammation, tissue repair, and wound chronicity, thereby prolonging healing time and increasing the risk of recurrence. In contrast to findings from gut microbiome research, where a high microbial load and diversity are generally associated with health, a lower bacterial burden appears to be more beneficial for skin health. While specific pathogens such as *P. aeruginosa, P. mirabilis*, and anaerobes have been repeatedly implicated in delayed or non-healing wounds, potentially protective genera, such as *Corynebacterium*, suggest a nuanced role of commensals and potentially beneficial modulation of pathogenic virulence. Anatomical location is significantly related to the wound microbiome, with the sacral region showing strong associations with *E. coli*, *Klebsiella* spp., and *S. aureus*, all linked to delayed healing. Larger wounds healed more slowly, and more severe wounds (grades III and IV) showed increased microbial diversity, with hard-to-heal wounds displaying particularly distinct microbiome profiles. Molecular diagnostics, including NGS, offer a standardised method and promising advances in revealing the dynamic microbial ecosystems of PIs and enable more precise interventions. Nevertheless, methodological heterogeneity, small study samples, and moderate bias in the available research limit robust conclusions. To optimise PI care, future investigations should prioritise standardised, patient-centred methodologies, wound care management and explore the contribution of the host immune response and microbiome interactions to the healing outcomes.

## Data Availability

The original contributions presented in the study are included in the article/**Supplementary Material**. Further inquiries can be directed to the corresponding author.
