# Supplementary material for: The association between pressure injury microbiome and wound healing: a systematic review
Source: Front Cell Infect Microbiol. 2026 Jan 8;15:1703418. doi: 10.3389/fcimb.2025.1703418 (PMC12823914; doi:10.3389/fcimb.2025.1703418)
Supplement: Supplementary file 2 [file DataSheet2.docx]

The influence of pressure injury microbiome on wound healing: a systematic review

E. Llukovi^1^, R. Wettstein^1,2^, E. Valido^1^, S. Capossela^1^, M. Gamba^3^, C. Peter^1^, J. Stoyanov^1,4^, A. Bertolo^1,4^*

| **Research Question Framework** | PEO |
| --- | --- |
| 1. Population | Humans |
| 2. Exposure | Microbiome in pressure injuries |
| 3. Outcome(s) | Ulcer Healing |

| **Information sources** | **Last date of search** | **Records retrieved** |
| --- | --- | --- |
| Embase.com | 01.10.24 | 1642 |
| Medline (R) (Ovid) | 01.10.24 | 737 |
| Web of Science Core Collection | 01.10.24 | 709 |
| Total before deduplication |  | 3088 |
| **Total after deduplication** |  | 2037 |

| **Sentinel papers (PMID & title)** | |
| --- | --- |
| 32249804 | The cutaneous microbiome in hospitalized patients with pressure ulcers |
| 32322583 | Distinct Skin Microbiome and Skin Physiological Functions Between Bedridden Older Patients and Healthy People: A Single-Center Study in Japan |
| 34531517 | Pressure ulcers microbiota dynamics and wound evolution |
| 36344337 | Relationship between healing status and microbial dissimilarity in wound and peri-wound skin in pressure injuries |
| 37532801 | Understanding the impact of spinal cord injury on the microbiota of healthy skin and pressure injuries |
| No PMID | Bacteriology and Antimicrobial Sensitivity of Isolated Bacteria from  Pressure Ulcers After Spinal Cord Injury |

**Medline (R) (Ovid)**

| #1 | exp Bacteria/ OR exp Microbiota/ OR exp Biofilms/ | 1'643'912 |
| --- | --- | --- |
| #2 | (((archaea* OR bacter* OR microbial OR microorganism* OR microbiot*) ADJ3 (communit* OR communit* composition* OR communit* structure* OR cohabitation)) OR ((biofilm* OR bio film*) ADJ3 (growth OR prevention)) OR ((microbial OR bacterial skin OR cutaneous aerobic OR cutaneous OR skin bacterial OR skin) ADJ3 flora) OR ((bacter* OR microbiot* OR microbiome* OR micro biome* OR microflor*) ADJ3 skin) OR ((microbiome* OR micro biome* OR microbiot*) ADJ3 (cutaneous OR human OR wound)) OR bacter* OR eubacter* OR microbe* OR microorganism* behavio* OR microorganism* model OR microorganism* OR microbiot* OR microflor* OR micro biome* OR microbiome* OR microbial biome* OR bio film* OR biofilm*).ti,ab | 1'123'087 |
| #3 | 1 or 2 | 2'035'756 |
| #4 | exp pressure ulcer/ | 14'509 |
| #5 | (((decubit* OR decubus OR bed OR pressure) ADJ3 (sore* OR ulcer* OR ulcus)) OR bedsore* OR pressure injur* OR pressure necros* OR decubitus OR chronically denuded skin).ti,ab | 19'111 |
| #6 | 4 or 5 | 22'528 |
| #7 | 3 and 6 | 819 |
| #8 | 7 not (exp animals/ not humans/) not ((exp infant/ or exp child/ or adolescent/) not (exp adult/)) | 737 |

**Web of Science Core Collection**

| #1 | TS=(((archaea* OR bacter* OR microbial OR microorganism* OR microbiot*) ADJ3 (communit* OR communit* composition* OR "communit* structure*" OR cohabitation)) OR ((biofilm* OR "bio film*") ADJ3 (growth OR prevention)) OR ((microbial OR "bacterial skin" OR "cutaneous aerobic" OR cutaneous OR "skin bacterial" OR skin) ADJ3 flora) OR ((bacter* OR microbiot* OR microbiome* OR "micro biome*" OR microflor*) ADJ3 skin) OR ((microbiome* OR "micro biome*" OR microbiot*) ADJ3 (cutaneous OR human OR wound)) OR bacter* OR eubacter* OR microbe* OR microorganism* behavio* OR microorganism* model OR microorganism* OR microbiot* OR microflor* OR "micro biome*" OR microbiome* OR "microbial biome*" OR "bio film*" OR biofilm*) | 1'854'313 |
| --- | --- | --- |
| #2 | TS=(((decubit* OR decubus OR bed OR pressure ) NEAR/3 (sore* OR ulcer* OR ulcus )) OR bedsore* OR "pressure injur*" OR "pressure necros*" OR decubitus OR "chronically denuded skin") | 19'982 |
| #3 | #1 AND #2 | 765 |
| #4 | TS=(((animal* OR rat OR rats OR mouse OR mice OR rodent OR squirrel OR murine OR nonhuman* OR primate*) NOT (human* OR patient* OR women OR woman OR men OR man))) | 3'904'870 |
| #5 | #3 NOT #4 | 716 |
| #6 | DT=(“Conference Abstract” OR Letter OR Note OR Editorial OR Preprint) | 4'100'271 |
| #7 | #5 NOT #6 | 709 |

**Embase.com Database**

| #1 | 'bacterium'/de OR 'microorganism'/de OR 'microflora'/exp OR 'microbiome'/exp OR 'human microbiome'/exp OR 'microbial community'/exp OR 'biofilm'/de OR 'skin flora'/exp OR 'skin microbiome'/exp OR 'microbiota composition'/exp | 586'545 |
| --- | --- | --- |
| #2 | ((archaea* OR bacter* OR microbial OR microorganism* OR microbiot*) NEAR/3 (communit* OR 'communit* composition*' OR 'communit* structure*' OR cohabitation)) OR ((biofilm* OR bio-film*) NEAR/3 (growth OR prevention)) OR ((microbial OR 'bacterial skin' OR 'cutaneous aerobic' OR cutaneous OR 'skin bacterial' OR skin) NEAR/3 (flora)) OR ((bacter* OR microbiot* OR microbiome* OR micro-biome* OR microflor*) NEAR/3 (skin)) OR ((microbiome* OR micro-biome* OR microbiot*) NEAR/3 (cutaneous OR human OR wound)) OR bacter* OR eubacter* OR microbe* OR 'microorganism* behavio?r' OR 'microorganism* model' OR microorganism* OR microbiot* OR microflor* OR micro-biome* OR microbiome* OR 'microbial biome*' OR bio-film* OR biofilm*:ti,ab | 2'436'907 |
| #3 | #1 OR #2 | 2'458'570 |
| #4 | 'decubitus'/exp | 28'680 |
| #5 | ((decubit* OR decubus OR bed OR pressure) NEAR/3 (sore* OR ulcer* OR ulcus)) OR bedsore* OR 'pressure injur*' OR 'pressure necros*' OR decubitus OR 'chronically denuded skin':ti,ab | 39'307 |
| #6 | #4 OR #5 | 39'307 |
| #7 | #3 AND #6 | 2'116 |
| #8 | #7 NOT ([animals]/lim NOT [humans]/lim) NOT ([conference abstract]/lim OR [letter]/lim OR [note]/lim OR [editorial]/lim) | 1'642 |

**Embase test with sentinel articles**

| #9 | 'bacteriology and antimicrobial sensitivity of isolated bacteria from pressure ulcers after spinal cord injury':ti | 1 |
| --- | --- | --- |
| #10 | #8 AND #9 | 1 |
| #11 | 'The cutaneous microbiome in hospitalized patients with pressure ulcers':ti | 1 |
| #12 | #8 AND #11 | 1 |
| #13 | 'Distinct Skin Microbiome and Skin Physiological Functions Between Bedridden Older Patients and Healthy People: A Single-Center Study in Japan':ti | 1 |
| #14 | #8 AND #13 | 1 |
| #15 | 'Pressure ulcers microbiota dynamics and wound evolution':ti | 1 |
| #16 | #8 AND #15 | 1 |
| #17 | 'Relationship between healing status and microbial dissimilarity in wound and peri-wound skin in pressure injuries':ti | 1 |
| #18 | #8 AND #17 | 1 |
| #19 | 'Understanding the impact of spinal cord injury on the microbiota of healthy skin and pressure injuries':ti |  |
| #20 | #8 AND #19 | 1 |

**Table S1.** Characteristics of studies included in the systematic review (n=23)

| **Characteristics** | **No. of studies** | **References** |
| --- | --- | --- |
| Study population |  |  |
| SCI | 8 | Andrianasolo, 2018; Binsuwaidan, 2023; Dunyach-Remy, 2021; Fazel, 2019; Lichtenthäler, 2023; Sapico, 1986; Singh, 2015; Wettstein, 2023 |
| Cancer | 1 | Sopata, 2002 |
| Hospitalized | 11 | Arisandi, 2020; Brook, 1991; Daltrey, 1981; Goh, 2021; Kunimitsu, 2023; Nagase, 2020; Sato, 2018; Sipponen, 2008; Shibata, 2021; de Wert, 2020; Yamashita, 2023 |
| Not reported | 0 |  |
| Sex |  |  |
| Male only | 1 | Wettstein, 2023 |
| Both | 22 |  |
| Female only | 0 |  |
| Not reported | 0 |  |
| Study size |  |  |
| <50 | 12 | Arisandi, 2020; Dunyach-Remy, 2021; Goh, 2021; Kunimitsu, 2023; Sapico, 1986; Singh, 2015; Sipponen, 2008; Shibata, 2021; Sopata, 2002; de Wert, 2020; Wettstein, 2023; Yamashita, 2023 |
| 50-100 | 6 | Andrianasolo, 2018; Brook, 1991; Daltrey, 1981; Fazel, 2019; Nagase, 2020; Sato, 2018 |
| >100 | 2 | Binsuwaidan, 2023; Lichtenthäler, 2023; |
| Not reported | 0 |  |
| Age, (Mean) |  |  |
| <16 | 1 | Brook, 1991 |
| 16-40 | 4 | Binsuwaidan, 2023; Fazel, 2019; Sapico, 1986; Singh, 2015 |
| >40-65 | 7 | Andrianasolo, 2018; Dunyach-Remy, 2021; Lichtenthäler, 2023; Sato, 2018; Sopata, 2002; Wettstein, 2023; Yamashita, 2023 |
| >65 | 8 | Arisandi, 2020; Daltrey, 1981; Goh, 2021; Kunimitsu, 2023; Nagase, 2020; Sipponen, 2008; Shibata, 2021; de Wert, 2020 |
| Not reported | 0 |  |
| Location |  |  |
| Europe | 8 | Andrianasolo, 2018; Daltrey, 1981; Dunyach-Remy, 2021; Lichtenthäler, 2023; Sipponen, 2008; Sopata, 2002; Wettstein, 2023; de Wert, 2020; |
| North America | 1 | Brook, 1991; |
| South America | 0 |  |
| Asia | 10 | Arisandi, 2020; Binsuwaidan, 2023; Fazel, 2019; Goh, 2021; Kunimitsu, 2023; Nagase, 2020; Sato, 2018; Shibata, 2021; Singh, 2015; Yamashita, 2023 |
| Africa | 0 |  |

**Table S2.** Risk of bias evaluation for controlled intervention studies using the NIH tool.

| **Study** | **Design** | **1** | **2** | **3** | **4** | **5** | **6** | **7** | **8** | **9** | **10** | **11** | **12** | **13** | **14** | **Risk of bias^1^** |
| --- | --- | --- | --- | --- | --- | --- | --- | --- | --- | --- | --- | --- | --- | --- | --- | --- |
| Sipponen *et al.,* 2008 | RCT | y | y | y | n | n | y | n | n | y | y | y | y | y | y | 10/14 (0.71)  Moderate |
| Sopata *et al.,* 2002 | RCT | y | y | n | n | n | y | NR | n | y | y | y | n | y | y | 8/14 (0.57)  Moderate |

Abbreviations: Y, yes; N, No; CD, cannot determine; NA, not applicable; NR, not reported
^1^Risk of bias rating (Low (75-100%), Moderate (25-75%), or High (0-25%))

**Criteria used to assess risk of bias of controlled intervention studies:**

1. Was the study described as randomized, a randomized trial, a randomized clinical trial, or an RCT?
2. Was the method of randomization adequate (i.e., use of randomly generated assignment)?
3. Was the treatment allocation concealed (so that assignments could not be predicted)?
4. Were study participants and providers blinded to treatment group assignment?
5. Were the people assessing the outcomes blinded to the participants' group assignments?
6. Were the groups similar at baseline on important characteristics that could affect outcomes (e.g., demographics, risk factors, co-morbid conditions)?
7. Was the overall drop-out rate from the study at endpoint 20% or lower of the number allocated to treatment?
8. Was the differential drop-out rate (between treatment groups) at endpoint 15 percentage points or lower?
9. Was there high adherence to the intervention protocols for each treatment group?
10. Were other interventions avoided or similar in the groups (e.g., similar background treatments)?
11. Were outcomes assessed using valid and reliable measures, implemented consistently across all study participants?
12. Did the authors report that the sample size was sufficiently large to be able to detect a difference in the main outcome between groups with at least 80% power?
13. Were outcomes reported or subgroups analyzed prespecified (i.e., identified before analyses were conducted)?
14. Were all randomized participants analyzed in the group to which they were originally assigned, i.e., did they use an intention-to-treat analysis?

**Table S3.** Risk of bias evaluation for pre-post studies using the NIH tool.

| **Study** | **1** | **2** | **3** | **4** | **5** | **6** | **7** | **8** | **9** | **10** | **11** | **12** | **Risk of Bias^1^** |
| --- | --- | --- | --- | --- | --- | --- | --- | --- | --- | --- | --- | --- | --- |
| Goh *et al.*, 2021 | y | y | y | y | n | y | y | n | y | n | n | n | 7/12 (0.58) Moderate |

Abbreviations: Y, yes; N, No; CD, cannot determine; NA, not applicable; NR, not reported
^1^Risk of bias rating (Low (75-100%), Moderate (25-75%), or High (0-25%))

**Criteria used to assess risk of bias of pre-post study without control group:**

1. Was the study question or objective clearly stated?
2. Were eligibility/selection criteria for the study population prespecified and clearly describe
3. Were the participants in the study representative of those who would be eligible for the test/service/intervention in the general or clinical population of interest?
4. Were all eligible participants that met the prespecified entry criteria enrolled?
5. Was the sample size sufficiently large to provide confidence in the findings?
6. Was the test/service/intervention clearly described and delivered consistently across the study population?
7. Were the outcome measures prespecified, clearly defined, valid, reliable, and assessed consistently across all study participants?
8. Were the people assessing the outcomes blinded to the participants' exposures/interventions?
9. Was the loss to follow-up after baseline 20% or less? Were those lost to follow-up accounted for in the analysis?
10. Did the statistical methods examine changes in outcome measures from before to after the intervention? Were statistical tests done that provided p values for the pre-to-post change
11. Were outcome measures of interest taken multiple times before the intervention and multiple times after the intervention (i.e., did they use an interrupted time-series design)?
12. If the intervention was conducted at a group level (e.g., a whole hospital, a community, etc.) did the statistical analysis consider the use of individual-level data to determine effects at the group level?

**Table S4**. Risk of bias evaluation for observational cohort & cross-sectional studies using the NIH tool.

| **Study** | **Design** | **1** | **2** | **3** | **4** | **5** | **6** | **7** | **8** | **9** | **10** | **11** | **12** | **13** | **14** | **Risk of bias^1^** |
| --- | --- | --- | --- | --- | --- | --- | --- | --- | --- | --- | --- | --- | --- | --- | --- | --- |
| Andrianasolo *et al.,* 2018 | Cohort (retrospective) | y | y | NR | CD | n | y | y | y | y | y | y | n | y | y | 10/14 (0.71)  Moderate |
| Arisandi *et al.,* 2020 | Cohort (prospective) | y | y | n | y | n | y | y | y | y | y | y | n | n | y | 10/14 (0.71) Moderate |
| Binsuwaidan *et.al.,* 2023 | Cohort (retrospective) | y | y | NR | y | y | n | y | y | y | NR | y | n | y | y | 10/14 (0.71) Moderate |
| Brook *et al.,* 1991 | Cohort (retrospective) | y | y | NR | CD | n | y | y | y | y | n | y | n | y | CD | 8/14 (0.57)  Moderate |
| Daltrey *et al.*, 1981 | Cohort (prospective) | y | y | NR | y | n | y | y | y | CD | y | y | n | n | y | 9/14 (0.64)  Moderate |
| Dunyach-Remy *et al*., 2021 | Cohort (prospective) | y | y | n | y | y | y | y | y | y | y | y | NR | NR | y | 11/14 (0.78)  Low |
| Kunimitsu et al., 2023 | Cohort (prospective) | y | y | y | y | n | y | y | y | y | y | y | n | y | CD | 11/14 (0.78)  Low |
| Nagase *et.al*., 2020 | Cohort (prospective) | y | y | NR | n | n | y | y | y | y | n | y | n | NR | y | 8/14 (0.57) Moderate |
| Sapico *et al.,* 1986 | Cohort (prospective) | n | n | NR | y | n | y | y | y | y | y | n | n | y | CD | 7/14 (0.5)  Moderate |
| Shibata *et al.,* 2021 | Cohort (prospective) | y | y | NR | y | NR | y | y | n | y | y | y | n | NR | y | 9/14 (0.64) Moderate |
| Wettstein *et al.,* 2021 | Cohort (prospective) | y | y | NR | y | n | y | y | y | y | n | y | n | NR | y | 9/14 (0.64) Moderate |
| Yamashita *et al.*, 2023 | Cohort (retrospective) | y | y | NR | n | n | y | y | NA | y | CD | y | n | NR | n | 6/14 (0.42) Moderate |
| Fazel *et.al*., 2019 | Cohort (retrospective) | y | y | y | y | n | y | y | y | y | n | y | n | y | NR | 10/14 (0.71) Moderate |
| Sato *et al.,* 2018 | Cohort (retrospective) | y | y | NR | y | n | y | y | NA | y | n | y | n | NR | y | 8/14 (0.57)  Moderate |

Abbreviations: Y, yes; N, No; CD, cannot determine; NA, not applicable; NR, not reported
^1^Risk of bias rating (Low (75-100%), Moderate (25-75%), or High (0-25%))

**Criteria used to assess risk of bias for observational cohort & cross-sectional studies:**

1. Was the research question or objective in this paper clearly stated?

2. Was the study population clearly specified and defined?

3. Was the participation rate of eligible persons at least 50%?

4. Were all the subjects selected or recruited from the same or similar populations (including the same time period)? Were inclusion and exclusion criteria for being in the study prespecified and applied uniformly to all participants?

5. Was a sample size justification, power description, or variance and effect estimates provided?

6. For the analyses in this paper, were the exposure(s) of interest measured prior to the outcome(s) being measured?

7. Was the timeframe sufficient so that one could reasonably expect to see an association between exposure and outcome if it existed?

8. For exposures that can vary in amount or level, did the study examine different levels of the exposure as related to the outcome (e.g., categories of exposure, or exposure measured as continuous variable)?

9. Were the exposure measures (independent variables) clearly defined, valid, reliable, and implemented consistently across all study participants?

10. Was the exposure(s) assessed more than once over time?

11. Were the outcome measures (dependent variables) clearly defined, valid, reliable, and implemented consistently across all study participants?

12. Were the outcome assessors blinded to the exposure status of participants?

13. Was loss to follow-up after baseline 20% or less?

14. Were key potential confounding variables measured and adjusted statistically for their impact on the relationship between exposure(s) and outcome(s)?

**Table S5.** Risk of bias evaluation for case-control studies using the NIH tool.

| **Study** | **1** | **2** | **3** | **4** | **5** | **6** | **7** | **8** | **9** | **10** | **11** | **12** | **Risk of bias^1^** |
| --- | --- | --- | --- | --- | --- | --- | --- | --- | --- | --- | --- | --- | --- |
| Lichtenthäler *et al.,* 2023 | y | y | n | y | y | y | NR | y | y | y | n | y | 9/12 (0.75) Moderate |
| Singh *et al.,* 2015 | y | y | n | y | y | y | n | y | y | y | n | y | 9/12 (0.75) Moderate |
| de Wert *et al*., 2020 | y | y | n | y | y | y | NR | y | y | y | n | n | 8/12 (0.6) Moderate |

Abbreviations: Y, yes; N, No; CD, cannot determine; NA, not applicable; NR, not reported
^1^Risk of bias rating (Low (75-100%), Moderate (25-75%), or High (0-25%))

**Criteria used to assess risk of bias of case-control studies:**

1. Was the research question or objective in this paper clearly stated and appropriate?

2. Was the study population clearly specified and defined?

3. Did the authors include a sample size justification?

4. Were controls selected or recruited from the same or similar population that gave rise to the cases (including the same timeframe)?

5. Were the definitions, inclusion and exclusion criteria, algorithms or processes used to identify or select cases and controls valid, reliable, and implemented consistently across all study participants?

6. Were the cases clearly defined and differentiated from controls?

7. If less than 100 percent of eligible cases and/or controls were selected for the study, were the cases and/or controls randomly selected from those eligible?

8. Was there use of concurrent controls?

9. Were the investigators able to confirm that the exposure/risk occurred prior to the development of the condition or event that defined a participant as a case?

10. Were the measures of exposure/risk clearly defined, valid, reliable, and implemented consistently (including the same time period) across all study participants?

11. Were the assessors of exposure/risk blinded to the case or control status of participants?

12. Were key potential confounding variables measured and adjusted statistically in the analyses? If matching was used, did the investigators account for matching during study analysis?
