# Supplementary material for: The association between pressure injury microbiome and wound healing: a systematic review
Source: Front Cell Infect Microbiol. 2026 Jan 8;15:1703418. doi: 10.3389/fcimb.2025.1703418 (PMC12823914; doi:10.3389/fcimb.2025.1703418)
Supplement: Supplementary file 3 [file DataSheet3.docx]

The influence of pressure injury microbiome on wound healing: a systematic review

E. Llukovi^1^, R. Wettstein^1,2^, E. Valido^1^, S. Capossela^1^, M. Gamba^3^, C. Peter^1^, J. Stoyanov^1,4^, A. Bertolo^1,4^*

**Table S6.** Pathogenicity of *Klebsiella*, *Staphylococcus*, *Proteus*, and *Pseudomonas*, which are commonly found in PIs.

| Bacterial species | Pathogenicity and Adverse Effects | Details |
| --- | --- | --- |
| *Klebsiella spp.* | Systemic Risk (Sepsis) | Superinfection of PIs with *Klebsiella* spp. may increase the risk of sepsis in patients with SCI. (Fazel^43^) |
|  | Antimicrobial Resistance | *Klebsiella* *pneumoniae* isolates are common multidrug-resistant (MDR) organisms, and Extended-Spectrum Beta-Lactamase (ESBL) producers are frequently found in PIs. (Binsuwaidan^19^) |
| *Staphylococcus spp*. | Impaired Cell Proliferation | *S. aureus* is negatively correlated with the Ki-67 index (a cell proliferation marker) in granulation tissue, demonstrating that high loads suppress granulation tissue formation and impair cell proliferation. (Sato^14^)  One study indicated that *S. aureus* retarded the healing process when it was the predominant flora. (Daltrey^38^) |
|  | Recurrence and Chronicity | A higher rate of *Staphylococcus* spp. on healed PI sites is associated with the development of recurrent PIs. (Shibata) |
|  | Deep Tissue and Systemic Infection | *S. aureus* is highly prevalent pathogen in PIs that progress to deep infections. (Wettstein^18^) *S. aureus* is a major contributor to PI-related pelvic osteomyelitis, often isolated in 47% of cases. (Binsuwaidan^19^, Andrianasolo^13^) It is also isolated in cases of bacteraemia. (Brook^37^, Fazel^43^) |
|  | Antimicrobial Resistance | *S. aureus* frequently exhibits Methicillin-Resistant S. aureus (MRSA). (Binsuwaidan^19^, Yamashita^42^) |
| *Proteus spp.* | Tissue Necrosis and Enlargement | *P. mirabilis* is strongly associated with necrotic and enlarging lesions. (Daltrey^38^) |
|  | Wound Degradation and Delayed Healing | It is commonly found in deep tissue cultures when the PI exhibits gross tissue necrosis. (Sapico^40^) The presence of *Proteus* spp. may contribute to local tissue damage, thereby delaying or preventing healing. (Daltrey^38^) |
|  | Association with Poor Evolution FEP | *Proteus* and *Morganella* genera were only present in wounds that had stagnated or worsened after 28 days in one study. These bacteria are part of a Functional Equivalent Pathogroup (FEP) that clusters together with strict anaerobic genera (*Anaerococcus* and *Peptoniphilus*). This FEP is frequently co-isolated from biopsies that have a poor evolution, suggesting a synergistic action that hinders healing. (Dunyach-Remy^16^) |
|  | Deep Infections | *P. mirabilis* is a prevalent species in sacral ulcer infections (Binsuwaidan^19^) and is a Gram-negative contributor to PI-related pelvic osteomyelitis. (Andrianasolo^13^) |

table continues the next page →

**Table S6.** Table continuation

| Bacterial species | Pathogenicity and Adverse Effects | Details |
| --- | --- | --- |
| *Pseudomonas spp.* | Tissue Damage and Biofilm Formation | Similar to *Proteus*, *P. aeruginosa* is strongly associated with necrotic and enlarging lesions. (Daltrey^38^)  It can contribute to local damage, thereby delaying or preventing healing. (Daltrey^38^) |
|  | Severe Complications | It is frequently isolated from infected PIs with concurrent soft tissue abscesses and osteomyelitis. In cases of pelvic osteomyelitis, *P. aeruginosa* superinfection was associated with high comorbidity. (Andrianasolo^13^) |
|  | Antimicrobial Resistance | *P. aeruginosa* is a Gram-negative species associated with MDR infections. (Binsuwaidan^19^) |
|  | Impact on Cell Proliferation (Contrasting View) | While high bacterial load generally suppresses healing, the quantity of *P. aeruginosa* specifically was not significantly correlated with the Ki-67 index (cell proliferation marker) in one study, suggesting its contamination may not directly disturb cell proliferation in the granulation tissue in the same manner as *S. aureus*. (Sato^14^) |

**Table S7.** Comparison of characteristics related to PI based on patient groups.

| Characteristic | SCI Patients | Elderly/Geriatric/Bedridden Patients |
| --- | --- | --- |
| Sources | Andrianasolo^13^, Binsuwaidan^19^, Dunyach-Remy^16^, Fazel^43^, Lichtenthäler^34^, Sapico^40^, Singh^17^, Wettstein^18^ | Arisandi^36^, Daltrey^38^, Kunimitsu^39^, Nagase^23^, Shibata^41^ |
| Typical Age Range | Predominantly younger adults in many studies, ranging from 30s to 60s. | Majority of PIs occur in old age (60–80 years of age). Studies focus on bedridden older patients (median age 85 years) or geriatric patients (mean age 79 years). |
| Primary Aetiology | PI results from unrelieved pressure coupled with loss of protective sensory perception (denervation), compounded by high risk of trauma-related injury. | PI results from prolonged immobility (bedridden/chair-bound), aging, and comorbidities. |
| Common PI Location | Pelvic region is highly dominant, including sacral (e.g., 56% in one study), ischial (28% in another), and trochanteric areas. | Generally PIs develop over bony prominences; sacral, ischial, and trochanteric. Sacral region is a frequent site for PIs in bedridden older patients. |
| Microbial Environment (Skin) | High likelihood of Gram-negative colonization of the perineum influenced by neurogenic bladder dysfunction, bacteriuria, and external catheter use. | Marked skin dysbiosis characterized by significantly higher pH on sacral skin, decreased commensal bacteria (e.g., *Cutibacterium* spp.), and a higher abundance of gut-related bacteria (*Escherichia–Shigella* spp., *Enterococcus* spp.). |
| Microbial Load (CFU Density) | In deep tissue with gross necrosis, the mean quantitative bacterial load was high: 6.4log_10_/g of tissue. In chronic ulcers, the average number of isolates per culture ranged from 1.25 to 3.3. | In superficial PIs of geriatric cancer patients, bacterial counts varied widely, from 1×10^2^ to 5×10^9^/g. Persistent colonization greater than 10^4^/cm^2^ is considered infection in geriatric lesions. |
| Prevalent Resistance | High prevalence of Multidrug-Resistant (MDR) organisms, including MRSA (up to 25.6% of isolates) and ESBL Enterobacteriaceae (17.7% of isolates). | Generally high risk for MDR organisms due to frequent hospital stays. |
| PI Progression/ Severity | High risk of severe complications: Stage 4 lesions are frequently associated with deep infections, including osteomyelitis (reported in 17%–32% of cases). | Often characterized by chronicity and high levels of inflammation. |
| Recurrence Risk Factors | Risk of recurrence (after surgical treatment/flap coverage) is associated with the existence of a previous PI at the same site. | High recurrence rate (26.7% within 6 weeks) seen after conservative treatment. Recurrence is significantly associated with elevated pH and the presence of *Acinetobacter* spp. on the healed skin, or Lower skin hydration and a high rate of Staphylococcus species. |
| Treatment Outcomes (Surgical/Healing Time) | Flap reconstruction failure rates vary; a study of PI-related pelvic osteomyelitis showed a 23% failure rate.  Median healing time for cured anus-near ulcers was 57.0 days (controls) to 75.5 days (faecal diversion group). | Grade II ulcers are reported to heal in 1–2 weeks, while Grade III ulcers take 6–12 weeks.  In one study of severe PIs (Grade II–IV) in geriatric patients, the final outcome was 94% fully healed in the resin salve group compared to 36% fully healed in the control group over 6 months. |
